# Late development of OCD-like phenotypes in *Dlgap1* knockout mice

**DOI:** 10.1007/s00213-024-06668-9

**Published:** 2024-08-23

**Authors:** Kimino Minagawa, Takashi Hayakawa, Hayato Akimoto, Takuya Nagashima, Yasuo Takahashi, Satoshi Asai

**Affiliations:** 1 Division of Genomic Epidemiology and Clinical Trials, Clinical Trials Research Center, Nihon University School of Medicine, 30-1 Oyaguchi-Kamicho, Itabashi-ku, Tokyo, 173-8610 Japan; 2 Division of Pharmacology, Department of Biomedical Sciences, Nihon University School of Medicine, Tokyo, Japan

**Keywords:** Obsessive compulsive and related disorders, Grooming and scratching behavior, Discs large associated protein, Knockout mouse, Serotonin-selective reuptake inhibitor

## Abstract

**Rationale:**

Despite variants in the *Dlgap1* gene having the two lowest *p*-value in a genome-wide association study of obsessive compulsive disorder (OCD), previous studies reported the absence of OCD-like phenotypes in *Dlgap1* knockout (KO) mice. Since these studies observed behavioral phenotypes only for a short period, development of OCD-like phenotypes in these mice at older ages was still plausible.

**Objective:**

To examine the presence or absence of development of OCD-like phenotypes in *Dlgap1* KO mice and their responsiveness to fluvoxamine.

**Methods and results:**

Newly produced *Dlgap1* KO mice were observed for a year. Modified SHIRPA primary screen in 2-month-old homozygous mutant mice showed only weak signs of anxiety, stress conditions and aggression. At older ages, however, these mutant mice exhibited excessive self-grooming characterized by increased scratching which led to skin lesions. A significant sex difference was observed in this scratching behavior. The penetrance of skin lesions reached 50% at 6–7 months of age and 90% at 12 months of age. In the open-field test performed just after the appearance of these lesions, homozygous mutant mice spent significantly less time in the center, an anxiety-like behavior, than did their wild-type and heterozygous littermates, none and less than 10% of which showed skin lesions at 1 year, respectively. The skin lesions and excessive self-grooming were significantly alleviated by two-week treatment with fluvoxamine.

**Conclusion:**

Usefulness of *Dlgap1* KO mice as a tool for investigating the pathogenesis of OCD-like phenotypes and its translational relevance was suggested.

**Supplementary Information:**

The online version contains supplementary material available at 10.1007/s00213-024-06668-9.

## Introduction

Obsessive compulsive disorder (OCD) is characterized by persistent and recurrent thoughts (obsessions) and/or repetitive behaviors aimed at reducing anxiety (compulsions), and is classified into a wider category of disorder, “obsessive-compulsive and related disorders (OCRD)”, in DSM-5 and ICD-11, together with phenomenologically and etiologically related disorders (American Psychiatric Association 2013; World Health Organization 2019). The pathogenesis of OCRD has remained largely unknown, but is believed to involve strong genetic contributions as well as some socio-environmental contributions (Monzani et al. 2014). While human genetic studies have revealed polygenic contributions, they also found variants in several genes to show relatively strong associations with OCRD (Gazzellone et al. 2016; Stewart et al. 2013). As a protein coded by such a gene, discs large associated protein (DLGAP; also known as SAPAP), a scaffolding protein that constitutes postsynaptic density of synapses, has attracted the attention of researchers. Among the five known isoforms of DLGAP, DLGAP1 to 4 are highly expressed across the brain with spatial patterns that partly overlap, and their genetic variants have been reported to be associated with various neuropsychiatric disorders including OCRD (Bai et al. 2022; Rasmussen et al. 2017).

The finding of associations between DLGAP and neuropsychiatric disorders encouraged studies of *Dlgap1* to *4* knockout (KO) mice, which resulted in the observation of behavioral phenotypes reminiscent of the neuropsychiatric disorders associated with each of these genes. Although usefulness of these KO mice as animal models was suggested, discrepancy between the results of previous studies of human genetic variants and KO mice can also be pointed out. While a genome-wide association study (GWAS) of OCD patients showed associations with two variants of *Dlgap1* to have the lowest, subthreshold *p*-values (Stewart et al. 2013; Wu et al. 2013), *Dlgap1* KO mice exhibited only impaired sociability and altered learning performance, but neither OCD-like nor anxiety-like behavior at 2 to 4 months of age in previous studies (Coba et al. 2018). This discrepancy contrasted with the observation of disrupted self-grooming and anxiety-like behavior of mice with knockout of *Dlgap3* (Welch et al. 2007), whose variants were significantly associated with OCRD in previous candidate-gene analyses (Bienvenu et al. 2009; Boardman et al. 2011; Crane et al. 2011; Züchner et al. 2009), but less significantly than those of *Dlgap1* in the GWAS. The combination of disrupted self-grooming and anxiety-like behavior has been considered to be an OCD-like phenotype, which is apparently similar to two disorders in the category of OCRD, trichotillomania (hair-pulling disorder) and excoriation (skin picking) disorder (Camilla d’Angelo et al. 2014; Feusner et al. 2009). In the present study, we addressed the above discrepancy, investigating the hypothesis that *Dlgap1* KO mice develop OCD-like phenotypes at older ages. This hypothesis warrants investigation, given the fact that the development of OCD-like phenotypes in two previously suggested animal models took several months to a year after birth (Manning et al. 2021; Ullrich et al. 2018), while other animal models exhibited much earlier development of OCD-like phenotypes (Casarotto et al. 2018; Shmelkov et al. 2010; Chen et al. 2010; Welch et al. 2007). Identifying the presence or absence of OCD-like phenotypes in older *Dlgap1* KO mice is expected to lay a foundation for further translational investigation of animal models to understand the broad spectrum of OCRD and its pathogenesis.

## Methods

### Animals

**Fig. 1:**
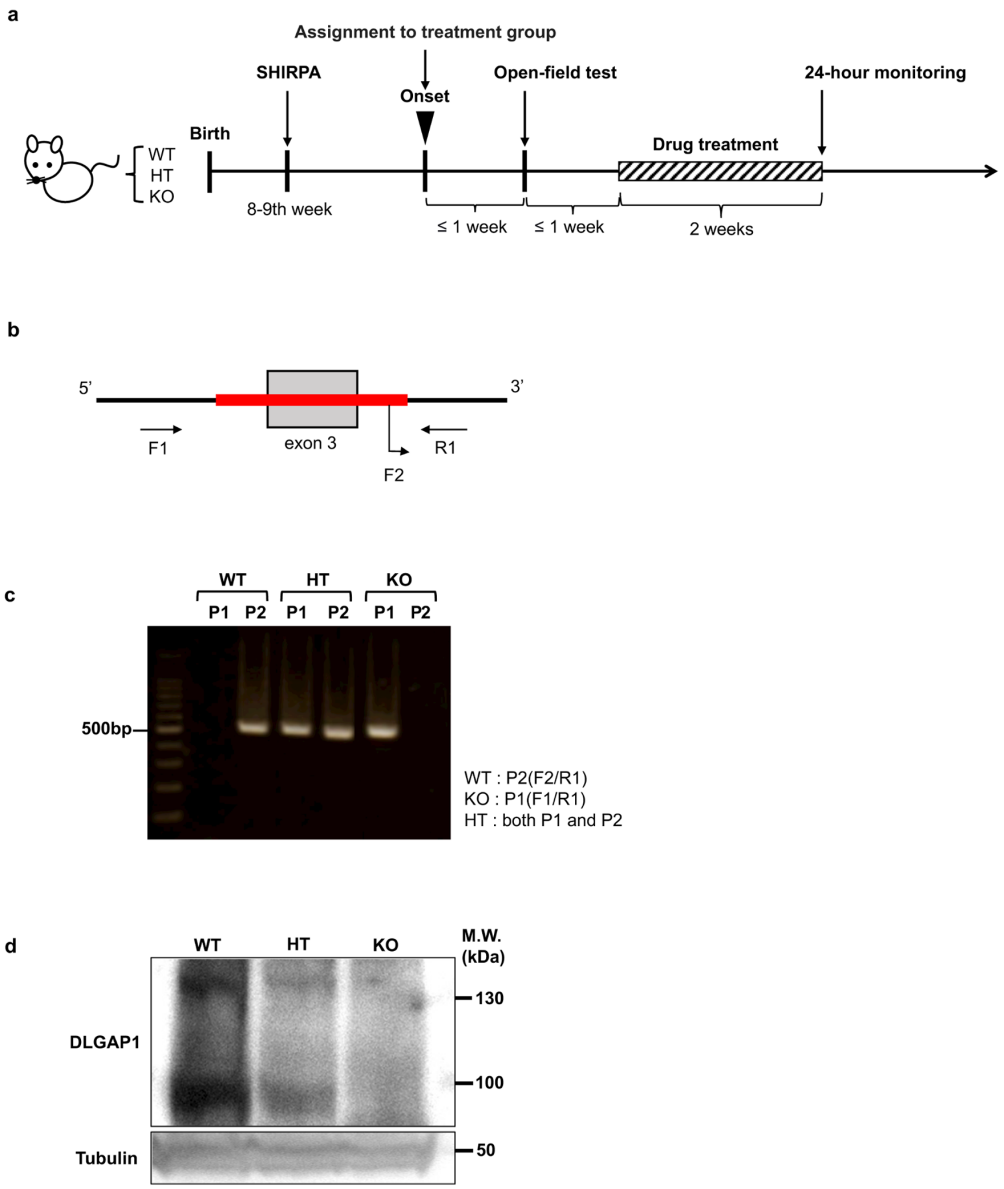
Experimental scheme and generation of knockout mice. (**a**) Timeline of observation for skin lesions and behavioral tests. Each triplet of KO, HT and WT littermates was longitudinally observed for skin lesions. At 2 months of age, mice were subjected to modified SHIRPA. If we observed skin lesions with score 2 in the KO mouse (i.e., onset), all members of the triplet were subjected to an open-field test, random assignment to a treatment group, two-week administration of vehicle or fluvoxamine, and 24-hour monitoring of self-grooming behavior, according to the schedule illustrated in the panel. (**b**) Knockout region and primers for genotyping. Red line indicates the knockout region. Forward_2 (F2) and reverse_1 (R1) primers that bind to the regions indicated by the corresponding arrows were used to detect the wild-type allele. Similarly, forward_1 (F1) and R1 primers were used to detect the knockout allele. (**c**) PCR analysis of mouse tail DNA with either of the two pairs of primers (P1: F1/R1 or P2: F2/R1). One of the following three patterns was detected to determine a genotype: WT, detection of a 513 bp band with P2; KO, detection of a 521 bp band with P1; HT, detection of both bands. (**d**) Immunoblots of brain synaptosome fraction probed with anti-DLGAP1 antibody. Specific bands of known sizes (approximately 100 kDa and 130 kDa: Rasmussen et al., 2017) were detected for the samples from WT and HT mice, but not for the sample from a KO mouse. β-tubulin was used as a loading control.

*Dlgap1* knockout mice with a C57BL/6N genetic background newly produced by Cyagen Biosciences (Guangzhou, China) were obtained. In the production of these KO mice, exon 3 of *Dlgap1* was deleted by CRISPR/Cas9-mediated genomic engineering. Heterozygous mutant mice were mated to produce *Dlgap1*^*−/−*^ (KO), *Dlgap1*^*+/−*^ (HT) and *Dlgap1*^*+/+*^ (WT) littermates. The littermates produced from each pair were separated by sex and then housed together (except for periods noted otherwise below) in standard animal cages containing Shepherd’s™ Cob (Shepherd Specialty Papers, Watertown, TN)-based bedding with a 12-hour light/dark cycle (lights on between 8:00 and 20:00) at room temperature of 23 ± 2 °C and relative humidity of 55 ± 5%, and they were fed ad libitum with standard laboratory chow (Oriental MF, Oriental Yeast Co., Tokyo) and water. From each animal cage, a triplet of KO, HT and WT littermates was selected, if possible, and used for further evaluation (see the timeline shown in Fig. 1a). The same single investigator observed the whole bodies of all of these triplets for skin lesions every day for one year except for weekends and public holidays. Severity of skin lesions were scored either 0 (no lesion), 1 (hairless patch/closed wound) or 2 (open wound). Four triplets in each sex for an initial pilot study were used for only skin observation. At two months of age, all other triplets were subjected to modified SHIRPA primary screen. Then, as soon as we observed skin lesions with score 2 in the KO mouse in a triplet, all members of the triplet were individually housed and subjected to an open-field test. Lesions with score 1 were rarely observed and always developed into score 2 within several days. This was followed by random assignment to a treatment group and two-week administration of vehicle or fluvoxamine, followed by 24-hour cage monitoring, as described below. The experimental protocol was approved by the ethics committee for animal experimentation of Nihon University School of Medicine (approval number: AP20MED064/067, AP22MED042/061, AP23MED015).

### Genotyping

All steps of the biochemical experiments in the present study were performed on ice, unless noted otherwise. Mice genotypes were identified by PCR analyses of DNA harvested from the tails of the mice on postnatal day 21 using a MightyAmp Genotyping Kit (Takara bio, Tokyo, Japan), with the following three primers: 5’-ACTTCTTGAAGGATGAAGCTACTGA-3’ (forward_1); 5’-TTTGCTGATCTCAGAGCTATCTTC-3’ (forward_2); 5’-GTGTCTCTGGCTACACAACATCT-3’ (reverse_1), as illustrated in Fig. 1b.

### Synaptic protein extraction

Mouse brains were homogenized in Syn-PER synaptic protein extraction reagent (Thermo Fisher Scientific, Waltham, MA, USA) mixed with EDTA-free protease inhibitor cocktail (FUJIFILM Wako Chemicals, Japan) using a Dounce tissue grinder. The homogenized tissue was centrifuged at 1,200 ×*g* for 10 min, and then the supernatant was centrifuged further at 15,000 ×*g* for 20 min. The pellets obtained by the second centrifugation (synaptosomes) were resuspended in Syn-PER reagent, and then stored at − 80ºC after their protein concentrations were measured using the Bradford method (Takara bio, Tokyo, Japan).

### Western blotting

Ten micrograms of harvested proteins were resolved with 4–12% SDS-PAGE gels and transferred onto PVDF membranes (Thermo Fisher Scientific, Waltham, MA, USA). Membranes were blocked with 5% non-fat dried milk in tris-buffered saline-0.1%/Tween-20 (TBS-0.1/T) for 1 h at room temperature, and then incubated overnight at 4ºC with either of the following primary antibodies: anti-DLGAP1 antibody (1:1000 dilution; GeneTex, Irvine, USA Cat#GTX133264) or anti-beta III tubulin antibody (1:1000 dilution; Abcam, Cambridge, UK; Cat# ab18207). Then, membranes were washed five times in TBS-0.05/T each for 5 min at room temperature. After incubation with secondary, goat anti-rabbit IgG-HRP antibody (1:5000 dilution; Proteintech, Wuhan, China Cat#SA00001-2) at room temperature for 1 h, membranes were washed with TBS-0.05/T. Immunoblots were developed with Amersham™ ECLTM Prime Western Blotting Detection Reagents (GE Healthcare, Little Chalfont, UK), and their chemiluminescence was detected using a LuminoGraph II imaging system (ATTO, Tokyo, Japan).

### SHIRPA primary screen (behavioral test battery)

The SHIRPA primary screen is a standardized protocol designed to provide a behavioral and functional profile of mice by observational assessment (Rogers et al. 1997). A modified version of SHIRPA (Masuya et al. 2005) was performed with 24 triplets of KO, HT and WT littermates (12 males and 12 females) at 8–9 weeks of age by the same single investigator with blinding to mouse genotypes, between 9:00 and 16:00. Complete details of the protocol are provided in the Official English webpage for RIKEN-modified SHIRPA (2012). Step 1 and 2 of this protocol were video-recorded for analysis from the side of the viewing jar and from right above the open-field arena, respectively. Prior to the test battery, mice were housed individually for 1 week. Then, mice were acclimatized to the testing room for 30 min just before the test battery. Using the 5-minute video-recordings of step 1 of the protocol performed in a transparent acrylate viewing jar with 10 cm inner diameter and 30 cm height, we also measured the number of grooming bouts and grooming time during this step (see next section for definition of these behavioral parameters).

### Open-field test

An open-field test of each triplet of KO, HT and WT littermates was performed within a week after (score-2) skin lesions were observed in the KO mouse (*n* = 10 for each combination of genotype and sex). Prior to the open-field test, each mouse was housed individually for a day, and then acclimatized to the testing room for 30 min just before the test. Then, the mouse was placed into the center of an open-field arena in a clear Plexiglas box (60 cm × 30 cm × 20 cm) with 18 (10 cm × 10 cm) squares drawn on its floor (Suppl. Figure 1a and b), and the mouse was allowed to explore for 5 min. Locomotor activity (number of square crossings), number of grooming bouts, grooming time, number of rearings, latency to leave the center and time spent in the center and in the periphery of the arena were determined manually by the same single investigator from a video-recording taken from right above the arena, with blinding to mouse genotypes.

A grooming bout was defined as a sequence of face-wiping, full-body grooming, rubbing of the head and ears and scratching behavior, according to Kalueff et al. (2007). Any complete or incomplete sequence of these behaviors delimited by lifting, licking, biting, or resting of a paw was counted as a single bout. The total time of these grooming bouts in a test was also measured. Rearing was defined as standing only on the hind limbs, with or without the forelimbs touching the wall of the arena.

### Treatment with fluvoxamine or vehicle

Mice examined in the open-field test were subsequently treated with vehicle (drinking water) or a serotonin-selective reuptake inhibitor (SSRI) (60 mg/kg/day fluvoxamine maleate dissolved in drinking water (FUJIFILM Wako Pure Chemicals, Japan)) for two weeks in cages for single mice. The amount of consumed liquid was measured at the time of changing the solution (once in two days) to ensure an appropriate dosage, and stable consumption of the prescribed dose was confirmed in all cases, except for relatively rare, occasional drops in consumption down to 48 mg/kg/day. In order to match the ages of the two treatment groups in each sex, just after the observation of skin lesions in each triplet of littermates, they were assigned to either the vehicle- or fluvoxamine-treated group in a pairwise random manner as follows. As we observed animals longitudinally, we paired each triplet with new onset of skin lesions with an unpaired triplet with previous onset, if the difference between the onset ages of the two triplets was within two weeks. Otherwise, we left the triplet as an unpaired one, and randomly assigned it to a treatment group. The triplet paired with this unpaired one later was assigned to the other treatment group. In this manner, we obtained five male and five female pairs of triplets for each combination of genotype and sex. After treatment for two weeks, the mice were video-recorded from the side of the same cages used for treatment, for 24 h in order to analyze their grooming behavior.

### 24-hour behavioral monitoring

In the analysis of recorded videos, the same single investigator blinded to sex, genotype and treatment group extracted scratching and non-scratching bouts according to the definition described below (see Lamothe et al. (2023) and literature cited therein for references) by manually checking animal behavior throughout the 24 h of a video-recording. Here, a scratching bout was defined as a sequence of behavior that starts by lifting of the hind limb to stroke toward the nape, head or body, and ends by placing the paw onto the floor or by biting or licking the claws. The definition of a non-scratching bout was similar to that of a grooming bout in the open-field test. Any complete or incomplete sequences of face-wiping, full-body grooming and rubbing of the head and ears, but not scratching, that lasted for more than 3 s in total were first extracted. Then, successive sequences separated by a period of less than 6 s were concatenated to form a single non-scratching bout, in order to avoid counting those interrupted by a short period of scratching as separate bouts. Note that such intervening scratching was not included in the concatenated non-scratching bouts, but dealt with separately. After these procedures, the numbers of scratching and non-scratching bouts were counted, and the total time spent on non-scratching bouts was measured. To see whether the choice of a 3-second threshold for detecting non-scratching bouts and a 6-second cutoff for their concatenation affected our conclusion, we performed the same analyses with 1 and 3-second thresholds and 1, 3 and 6-second cutoffs in four 30-minute segments (4:00–4:30, 10:00–10:30, 18:00–18:30 and 21:00–21:30) taken from the 24-hour video-recording for each mouse.

### Statistics

We performed two-way mixed ANOVA (and two-way ANCOVA) with suitably chosen factors (and covariates) for analysis of the results of SHIRPA and open-field test and analysis of the genotype effect (and treatment effect) in 24-hour monitoring, respectively, with Tukey post-hoc tests with Bonferroni corrections (Kassambara 2019). In these analyses, a triplet of KO, HT and WT littermates was regarded as a single subject, implying that genotype was used as a within-subject factor in order to control effects of unknown factors shared among littermates, while sex and treatment group were used as between-subject factors. We tested the hypotheses of normality and between-subject equi-(co)variance of data itself or residuals of linear regression in mixed ANOVA and ANCOVA, respectively, by performing Shapiro-Wilk test, Levene test and box-M test (with cutoffs, *p* = 0.05, 0.05 and 0.001, respectively). Greenhouse-Geisser sphericity correction was automatically applied by the software to within-subject factors violating the sphericity assumption (i.e., *p* < 0.05 in Mauchly’s test). For cases with either of these hypotheses rejected and for cases with integer-valued scores where normality could not be assumed, we performed mixed ANOVA with aligned rank transformation (ART) (Wobbrock et al. 2011) followed by post-hoc pairwise Wilcoxon rank-sum (or signed-rank) tests with Bonferroni corrections. For a significant interaction involving genotype, Friedmann test was performed for each level of the other factor before pairwise comparisons. ANCOVA for treatment effect on grooming was performed with several sets of covariates, and the set of covariates with the best evidence in terms of Bayesian information criterion (BIC) was employed. Before the main analysis in ANCOVA, we confirmed the homogeneity of regression slopes for covariates. For analysis of treatment effect on skin lesions, two-way ART-ANOVA of changes in skin-lesion scores was performed without covariates. For grooming-related behavioral parameters of KO mice in the open-field test, we performed *k*-means clustering after standardization of variance, identifying distinct grooming levels. Choice of number of clusters in this analysis was evaluated in terms of BIC, and variables representing thus obtained clusters were added to the set of candidate covariates for ANCOVA for testing treatment effects on grooming. To carry out the procedures described above, we used free software, R (RCoreTeam 2023), with cluster, coin, rstatix and ARTool packages. For the analysis of longitudinally-observed skin lesions, survival curves of mice with different sexes and genotypes were fitted using Kaplan-Meier estimators and compared by performing log-rank tests, using JMP Pro16 software (SAS Institute Inc., Cary, NC, USA). The data processed in the manner described above were plotted graphically using GraphPad Prism (version 8, San Francisco, La Jolla, California, USA).

## Results

### Confirmation of genotypes and expression of DLGAP1 in brain

The genotype of KO mice produced by deleting exon 3 (as illustrated in the schema of Fig. 1b) as well as those of their HT and WT littermates were confirmed on the basis of the results of PCR analysis of DNA harvested from the tails of these mice (Fig. 1c). By performing western blot analysis with homogenates of synaptosomes obtained from the brain of thus genotyped mice, we confirmed that KO mice lacked DLGAP1 protein (Fig. 1d).

### SHIRPA primary screen

Two-way mixed (ART-)ANOVA of the results of SHIRPA primary screen performed on mice at 2 months of age and post-hoc tests with Bonferroni corrections did not detect any significant morphological difference among three genotypes, nor was there any significant difference in behavioral parameters, including those for behavior directly or indirectly related to excessive self-grooming and anxiety, such as number of grooming bouts, grooming time, locomotor activity, latency to start moving, fear, irritability and aggression, except that the number of defecations (number of urinations and tail-elevation score) was significantly higher in KO and HT (only KO) mice, compared to WT littermates (defecation: KO vs. WT, *p* < 0.001, HT vs. WT, *p* < 0.001; urination: KO vs. WT, *p* = 0.047; tail-elevation score: KO vs. WT, *p* = 0.001), touch-escape score was significantly lower in KO mice compared to WT mice (*p* = 0.001), and weakly significant genetic effects were observed for body weight and body-mass index with apparently inconsistent results of post-hoc tests (body weight: KO vs. HT, *p* = 1.000; KO vs. WT, *p* = 0.942; HT vs. WT, *p* = 0.037, body-mass index: KO vs. HT, *p* = 0.031; KO vs. WT, *p* = 0.381, HT vs. WT, *p* = 0.354). On the other hand, sex differences without interaction with genotype were observed for body length, tail length, body weight and body-mass index. The details of the results of modified-SHIRPA are provided in Suppl. Table 1. Also see Suppl. Figure 5a and b for the details of grooming-related parameters.

### Skin lesions in KO mice

**Fig. 2:**
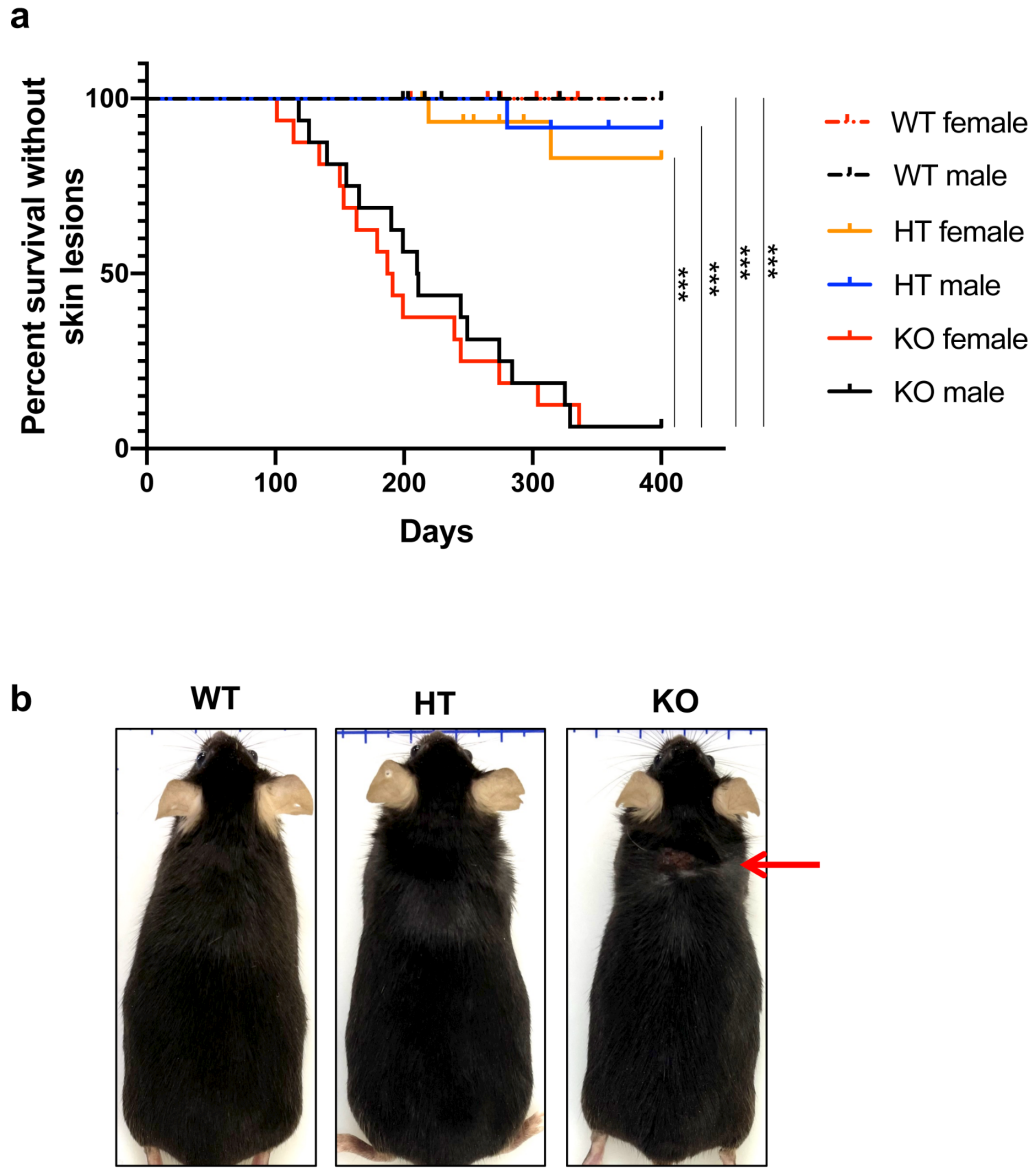
Occurrence of skin lesions. (**a**) Kaplan-Meier estimators for skin-lesion-free survival rate (with 16 mice for each combination of sex and genotype). Log-rank test was used to determine statistical significance of the difference between two curves (indicated by, ***p < 0.001; **p < 0.01; *p < 0.05) under Bonferroni correction. Comparison between genotypes (female: KO vs WT, p < 0.001; KO vs HT, p < 0.001; HT vs WT, p = 0.481, male: KO vs WT, p < 0.001; KO vs HT, p < 0.001; HT vs WT, p = 1.000) and sexes (female vs male: KO, p = 0.658; HT, p = 0.490; WT, p = 1.000) was performed. (**b**) Appearance of a representative triplet of KO, HT and WT littermates at the onset of skin lesions in the KO mouse. Skin lesions are indicated by a red arrow.

As early as 3 months of age, some KO mice developed score-2 skin lesions on their head, neck, face, snout or ears (Fig. 2b). In both sexes, the penetrance of this phenotype increased with age, reaching 50% around 6–7 months of age (Fig. 2a). At 12 months of age, 90% of KO mice were affected. Skin lesions progressed over time, and some KO mice eventually developed ulcers with hemorrhage. On the other hand, the incidence of skin lesions was 0% and about 10% at 12 months of age, in WT and HT littermates, respectively. Skin lesions were observed in KO mice, regardless of the genotypes of their co-housed partners.

### Excessive self-grooming and anxiety-like behavior in KO mice

**Fig. 3:**
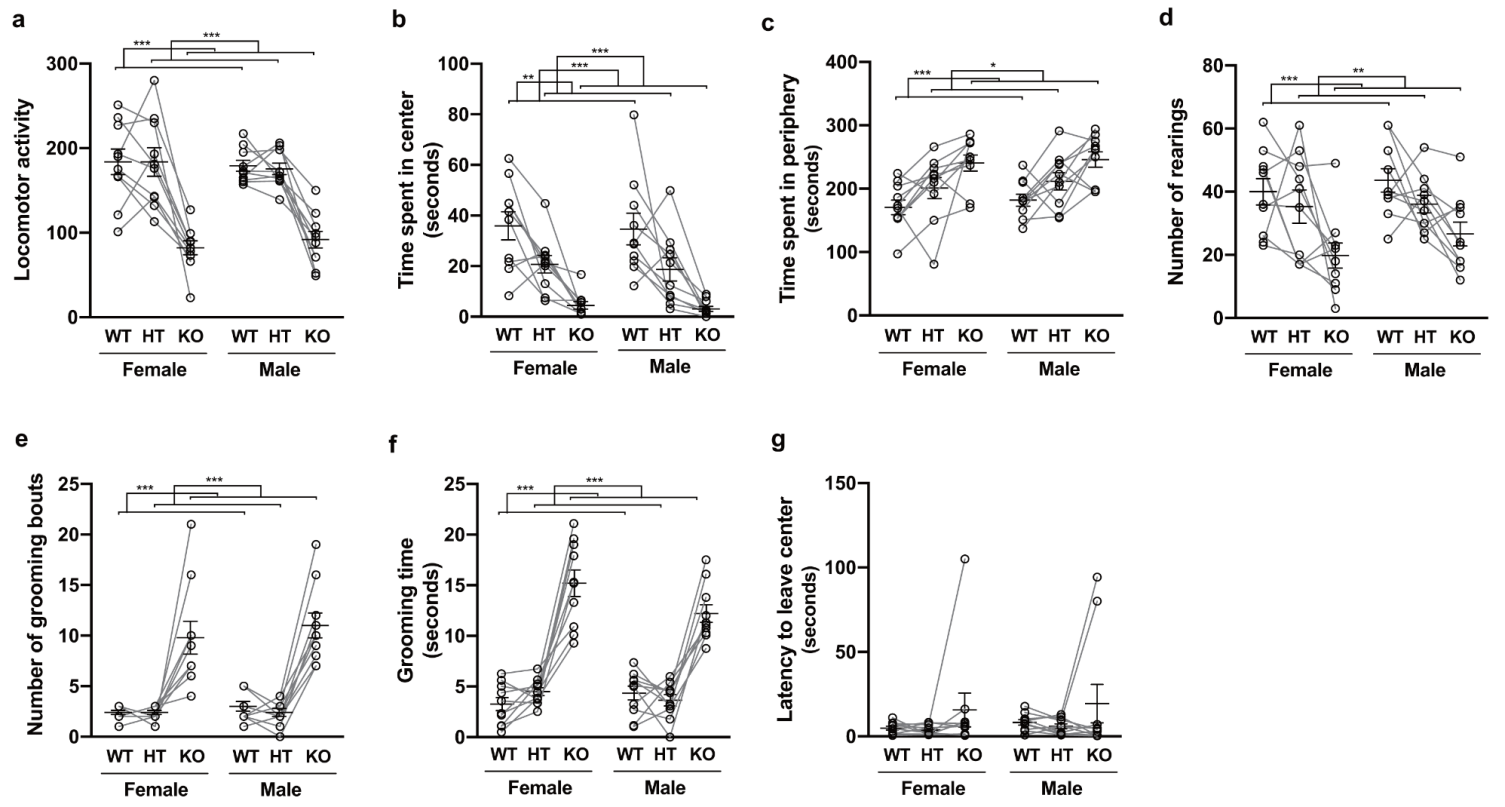
Results of open-field test. For each combination of genotype and sex, the mean and its standard error of the behavioral parameter indicated in each of panel (**a**)–(**g**) are plotted together with data points for individual mice and lines connecting data points for littermates. Data were analyzed by two-way mixed (ART-)ANOVA (using genotype and sex as factors). Statistically significant differences confirmed by post-hoc tests are indicated in the panels (***p < 0.001; **p < 0.01; *p < 0.05). Results that did not reach the significance level are not shown in the panel.

For KO mice exhibiting skin lesions and their littermates, an open-field test was performed within 1 week after the observation of the lesions. Two-way mixed (ART-)ANOVA of locomotor activity, time spent in the center and in the periphery of the arena, numbers of rearings and grooming bouts, and grooming time revealed a significant main effect of genotype (Fig. 3a–f: locomotor activity, *F*_2, 36_ = 67.27, *p* < 0.001; time spent in the center, *F*_2, 36_ = 48.72, *p* < 0.001; time spent in the periphery, *F*_2, 36_ = 18.01, *p* < 0.001; number of rearings, *F*_2, 36_ = 13.15, *p* < 0.001; number of grooming bouts, *F*_2, 36_ = 55.19, *p* < 0.001; grooming time, *F*_2, 36_ = 81.28, *p* < 0.001), whereas the main effect of sex and two-way interaction showed no significance in either analysis. Post-hoc tests further showed less locomotor activity, shorter time spent in the center, longer time spent in the periphery, fewer rearings, more grooming bouts, and longer grooming time of KO mice compared to those of their WT (Fig. 3a–f: *p* < 0.001, respectively) and HT littermates (Fig. 3a–f: time spent in the periphery, *p* = 0.025; number of rearings, *p* = 0.004; the others, *p* < 0.001, respectively). We also observed significant differences between HT and WT littermates in terms of time spent in the center (Fig. 3b: *p* = 0.008), but not in terms of the other behavioral parameters. In contrast with the behavioral parameters described above, two-way mixed ART-ANOVA of latency to leave the center revealed no significance in either the main effects of genotype and sex nor the two-way interaction, while we observed three KO mice that exhibited initial freezing as outliers (Fig. 3g). Complete information for the analysis described above is provided in Suppl. Table 2. Plots of these behavioral parameters (grooming parameters) with onset age (month) are provided in Suppl. Figure 2a–f (4a and b), where we found no significant (apparent) correlation.

### 24-hour monitoring and alleviation of excessive grooming and skin lesions by treatment

After the two-week treatment subsequent to the open-field test, we observed that skin lesions of all fluvoxamine-treated KO mice were almost completely healed, compared to the persistent lesions of their vehicle-treated pairs (Fig. 5h), as confirmed by two-way ART-ANOVA of changes in skin-lesion scores (Fig. 5g) with a significant main effect of treatment (*F*_1, 8_ = 200.0, *p* < 0.001). See Suppl. Table 6 for the details of the statistical analysis.

**Fig. 4:**
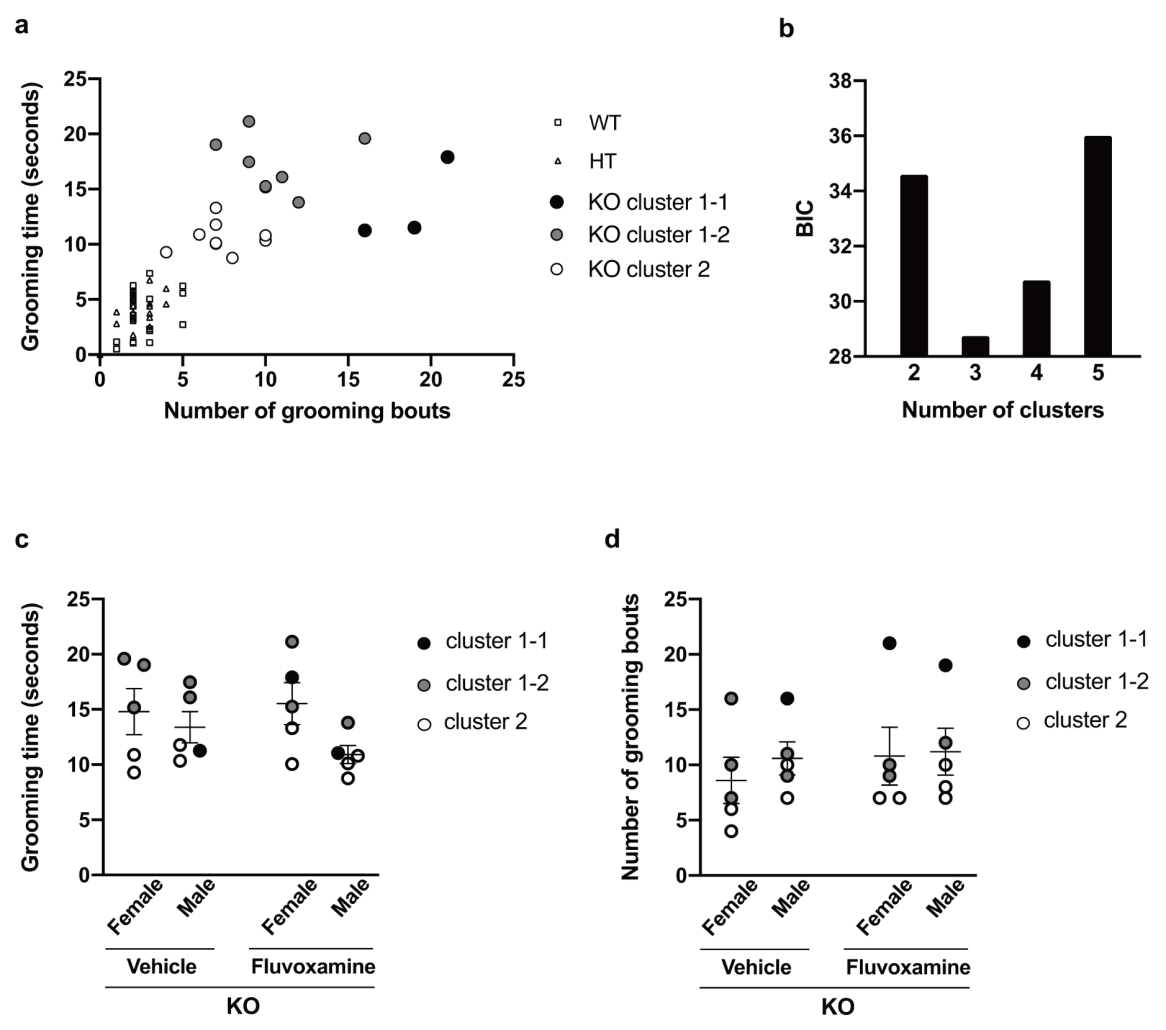
k-means clustering of results of open-field test. (**a**) Results of k-means clustering of (standardized) grooming-related behavioral parameters of KO mice in open-field test with assumption of two and three clusters are shown, together with those parameters for HT and WT mice. Analysis assuming two clusters detected cluster 1 and 2, while analysis assuming three clusters detected two subclusters 1-1 and 1-2 in cluster 1. (**b**) BIC values for k-means clustering with assumption of two to five clusters are shown. Grooming time (**c**) and number of grooming bouts (**d**) of KO mice are plotted together with sex, treatment group and results of clustering. No significant difference in these behavioral parameters across sexes and treatment groups was detected by mixed ART-ANOVA (Suppl. Table 10), and no dependence of the assigned cluster on the sex and treatment group was detected by Mantel-Haenszel and Woolf tests (Suppl. Table 11).

To see whether this treatment effect was associated with changes in grooming behavior, we measured numbers of scratching and non-scratching bouts and total time spent on non-scratching bouts in the 24-hour behavioral monitoring carried out after treatment, and performed ANCOVA of these behavioral parameters in KO mice using sex and treatment as factors. In this ANCOVA, we made adjustment for baseline grooming level using the results of the open-field test as covariates. Since grooming time and number of grooming bouts of KO mice in the open-field test were apparently heterogeneous, we performed *k*-means clustering of these results, identifying a few discrete grooming levels (Fig. 4a), and added these grooming levels to the set of covariates. To see whether the basic assumptions for ANCOVA hold, we also performed ART-ANOVA of the results of the open-field test with sex and treatment group as factors, confirming absence of significant dependence (Fig. 4c and d and Suppl. Table 10). Independence of discrete grooming level from treatment group across sexes was also confirmed by Mantel-Haenszel and Woolf tests (Suppl. Table 11).

**Fig. 5:**
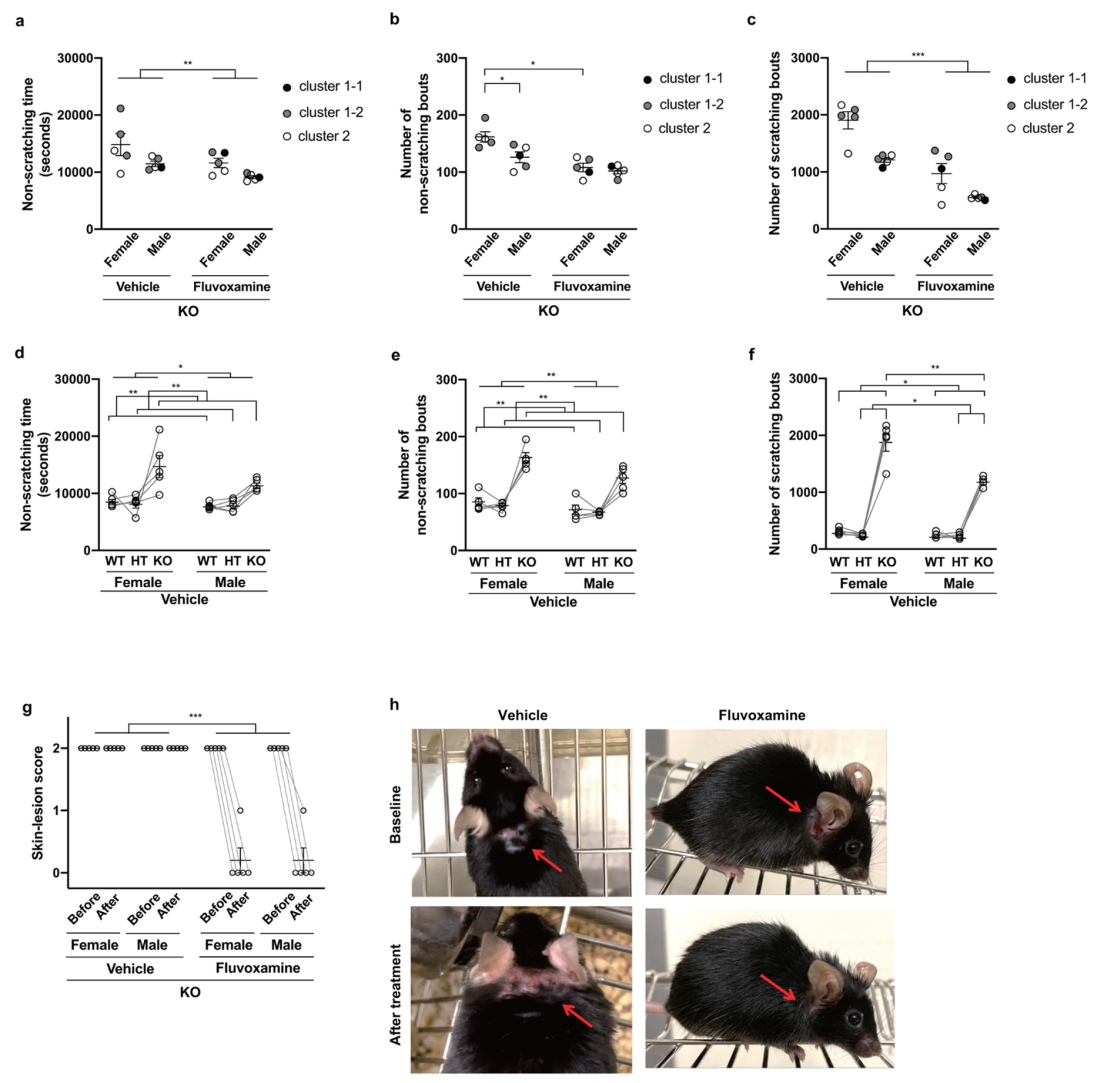
24-hour monitoring of grooming behavior after treatment and changes in skin lesions. For each combination of sex and treatment in KO mice, or for each combination of sex and genotype in vehicle-treated mice, the mean and its standard error of the behavioral parameter indicated in each of panel (**a**)–(**g**) are plotted together with data points for individual mice and lines connecting data points for littermates. In (**g**), both scores before and after treatment are shown. Data were analyzed by performing (**a**)–(**c**) two-way ANCOVA (using treatment and sex as factors), (**d**)–(**f**) two-way mixed ART-ANOVA (using genotype and sex as factors) or (**g**) two-way mixed ART-ANOVA of changes between before and after treatment (using treatment and sex as factors). Statistically significant differences confirmed by post-hoc tests are indicated in the panels (***p < 0.001; **p < 0.01; *p < 0.05). Results that did not reach the significance level are not shown. (**h**) Appearances of representative vehicle- and fluvoxamine-treated KO mice before and after treatment are shown, respectively. Skin lesions are indicated by red arrows.

After these pre-tests of assumptions in addition to those described in Methods, we performed ANCOVA of behavioral parameters measured in 24-hour monitoring of KO mice with different sets of covariates chosen among onset age, grooming time, number of grooming bouts and discrete grooming levels, and compared evidence in terms of BIC for multivariate linear regressions performed in ANCOVA (Suppl. Tables 3–5). Here, we used different numbers of discrete grooming levels, although the optimal number of clusters in *k*-means clustering was three (Fig. 4b). Note that optimal numbers of clusters in clustering and ANCOVA do not necessarily coincide. With the optimal set of covariates, two-way ANCOVA of number of non-scratching bouts detected a significant main effect of treatment (*F*_1, 11_ = 29.50, *p* < 0.001) and a significant interaction of sex and treatment (*F*_1, 11_ = 10.64, *p* < 0.001). Post-hoc analysis revealed significantly fewer bouts in fluvoxamine-treated females compared to vehicle-treated females (*p* = 0.021) and in vehicle-treated male compared to vehicle-treated females (*p* = 0.022) (Fig. 5b). For non-scratching time and number of scratching bouts, we performed one-way ANCOVA because of non-homogeneity of regression slopes for covariates between males and females, abandoning the analysis of sex differences and expanding the covariates with non-homogeneity into ones with sex-wise levels for which we did not detect non-homogeneity between treatment groups. Both analyses with optimal sets of covariates detected a significant main effect of treatment (non-scratching time, *F*_1, 12_ = 15.71, *p* = 0.002; number of scratching bouts, *F*_1, 12_ = 107.19, *p* < 0.001) with shorter non-scratching time and fewer scratching bouts in fluvoxamine-treated mice (Fig. 5a and c). Although a significant treatment effect described above was observed regardless of the choice of covariates, it is noteworthy that use of two baseline grooming levels as a covariate led to largely improved BIC values for non-scratching time and number of scratching bouts (Suppl. Tables 3 and 5), while use of three baseline levels led to the best BIC value for number of non-scratching bouts (Suppl. Table 4). The former could also be visually seen from the difference in the two behavioral parameters between females with two baseline levels (Fig. 5a and c).

Since we also measured behavioral parameters in HT and WT littermates in the 24-hour monitoring, we additionally compared behavioral parameters of vehicle-treated mice across different sexes and genotypes. Although non-normality was detected for all behavioral parameters, two-way mixed ART-ANOVA revealed significant main effects of genotype (non-scratching time, *F*_2, 16_ = 13.27, *p* < 0.001; number of non-scratching bouts, *F*_2, 16_ = 24.10, *p* < 0.001; number of scratching bouts, *F*_2, 16_ = 41.76, *p* < 0.001) and sex (non-scratching time, *F*_1, 8_ = 8.70, *p* = 0.018; number of non-scratching bouts, *F*_1, 8_ = 14.34, *p* = 0.005; number of scratching bouts, *F*_1, 8_ = 37.95, *p* < 0.001). A significant interaction between genotype and sex was detected for number of scratching bouts (*F*_2, 16_ = 16.79, *p* < 0.001), but not for the other parameters. For both non-scratching time and number of non-scratching bouts, post-hoc pairwise tests revealed significantly larger values in KO compared to HT (*p* = 0.006 for both) and WT (*p* = 0.006 for both) (Fig. 5d and e). For number of scratching bouts, pairwise tests revealed significantly smaller contrasts between KO and WT (HT) in males compared to females (vs. WT, *p* = 0.024; vs. HT, *p* = 0.048), but no significant difference between different genotypes of each sex (Fig. 5f), despite significant main effects of genotype in post-hoc Friedman tests for females (*p* = 0.007) and males (*p* = 0.022). The obtained *p*-values for the comparison of KO with HT and WT in each sex was the lowest possible value for our sample size, suggesting a shortage of samples.

Complete information of the analysis described in this section is provided in Suppl. Tables 3–7. Plots of the three behavioral parameters with onset month are provided in Suppl. Figure 4c–e, where we found no apparent correlation. The robustness of the treatment effect in KO mice and the genotype effect in vehicle-treated mice were confirmed with different cutoff/threshold values for the definition of non-scratching bout (Suppl. Figure 3a–p and Suppl. Tables 8 and 9).

## Discussion

In the present study, we observed skin lesions to develop at older ages in *Dlgap1* KO mice. The estimated penetrance of skin lesions among KO mice gradually increased with age, from the earliest occurrence in a 3-month-old mouse, and reached 50% at 6–7 months of age and 90% at 12 months of age. The clear difference between this development in KO mice and the rare occurrence in WT and HT littermates indicated a genetic effect.

At the onset of skin lesions, increased grooming behavior was observed in KO mice in the open-field test, compared to their HT and WT littermates. Subsequent 24-hour monitoring of grooming behavior divided into scratching and non-scratching bouts in vehicle-treated mice revealed marked and moderate increases in scratching and non-scratching behavior in KO mice, respectively. In this analysis, a sex difference in scratching behavior in KO mice compared to HT and WT littermates was also observed. Comparing skin lesions and grooming behavior in 24-hour monitoring between treatment groups, we further observed the administration of fluvoxamine for two weeks to alleviate skin lesions, reducing the frequency of self-grooming. In this analysis, the effects of genotype and treatment on non-scratching behavior were much less striking, compared to those on scratching behavior and those on self-grooming in previous studies without the distinction of scratching and non-scratching behavior (Welch et al. 2007). The same patterns of effects of genotype (and treatment) were observed in *Dlgap3* mutant mice by Lamothe et al. (2023) and Piantadosi et al. (2024). Notably, however, Piantadosi et al. (2024) also showed that excessive non-scratching grooming behavior, but not scratching behavior, was associated with central-striatal hyperactivity in KO mice whose optogenetic suppression resulted in reduced grooming.

In the open-field test performed just after the onset of skin lesions, KO mice exhibited more anxiety-like behavior as well, as indicated by the reduced time spent in the center. Although the reduced number of rearings exhibited by our KO mice is usually related to reduced anxiety (Bailey and Crawley 2009), this can also be interpreted as a consequence of proportionally reduced locomotor activity which might be related to freezing due to fear and anxiety (Miyata et al. 2007). Use of additional assays for anxiety-like behavior, such as elevated plus maze test and light-dark box test, would be helpful to support this interpretation in future studies. Interestingly, our HT mice spent less time in the center than did WT littermates, while they did not show significantly increased self-grooming in both of the open-field test and 24-hour monitoring. Noting that about 10% of HT mice exhibited skin lesions at 1 year of age, this difference might be interpreted as an early sign of the OCD-like phenotype, as similarly observed in human patients (Juckel et al. 2014). The causal relationship between anxiety and compulsion in OCD is controversial (Gillan and Robbins 2014), and it might therefore be interesting to investigate how these cognitive processes interact with each other through the slow development in our HT mice.

In fairness, in the interpretation of our results, we should mention a major limitation of our study design that we did not perform 24-hour monitoring before treatment and relied only on the results of the open-field test for controlling the level of baseline grooming behavior. This would have introduced additional noise originating from unmeasured variation in baseline grooming to the results. However, while available baseline grooming parameters in the open-field test showed strong correlations with the number of scratching bouts and non-scratching time in females, independence of treatment effects from these parameters could be seen from the homogeneity of regression slopes for covariates between treatment groups and also visually in Fig. 5a and c. The treatment effect on number of scratching bouts in male KO was much larger than the variation within treatment groups (Fig. 5c), and was also so in female KO after optimal adjustment for covariates, as seen from the strong significance of the treatment effect and the equivariance of residuals among groups of different sex and treatment (results of Levene test in Suppl. Table 5). The detected treatment effect on self-grooming was consistent with the clear treatment effect on skin lesions for which we observed pre-treatment scores. We therefore concluded that our evidence of a treatment effect on grooming behavior is sufficiently convincing.

On the basis of the results described above, the presence of abnormal self-grooming and anxiety-like behavior at older ages, which we initially hypothesized, was confirmed. The clear manifestations of behavioral phenotypes at older ages contrasted with the lack of significant differences in the scores of modified-SHIRPA performed at younger ages, in terms of both self-grooming (Suppl. Figure 5a and b) and anxiety-related behavior (latency to move, locomotor activity, fear, irritability and aggression), except for increased numbers of defecations and urinations, elevated tail position and reduced escape response to touch in KO mice (Suppl. Table 1). Among the significant differences, increased defecation and urination and elevated tail positions have been related to increased stress and anxiety (Swiergiel and Dunn 2006; Dityatev and Bolshakov 2005), and reduced escape response to touch might be related to freezing due to these conditions. These results of SHIRPA were consistent with a previous study of behavior in another strain of *Dlgap1* KO mice with almost the same genetic background (C57/BL6J: Coba et al. 2018) as ours, in which 2-month-old KO mice exhibited only weak signs of anxiety, stress and aggression. Taken together, we conclude that 2-month-old *Dlgap1* KO mice with C57/BL6 backgrounds do not show established OCD-like phenotypes, while weak signs of cognitive and behavioral alterations have been detected.

In comparison with previous studies, the time course of the development of skin lesions in our study was comparable with those in *Spred2* KO mice (Ullrich et al. 2018) and *Dlgap3* KO mice with C57/BL6J background (Manning et al. 2021), and was much longer than those in *Slitrk5*, *Hoxb8* and *iNos2* KO mice (Casarotto et al. 2018; Chen et al. 2010; Shmelkov et al. 2010) and another strain of *Dlgap3* KO mice (Welch et al. 2007) with a mixed 129/Sv/C57BL/6 background which was not shown in the original report, but was disclosed in a personal communication with Dr. Guoping Feng in Manning et al. (2019). In particular, our results were apparently similar to the relatively abrupt increase of abnormal self-grooming mostly accompanied by skin lesions in *Dlgap3* KO mice between 4 and 8 months with 50% penetrance at 8 months (Manning et al. 2021). Although the difference in the onset of abnormal self-grooming between the two strains of *Dlgap3* KO mice suggests involvement of unknown genetic or environmental factors, the large variability in onset among *Dlgap1* and *3* KO mice of the same strain in both our study and that of Manning et al. (2021) is also noteworthy.

Besides the classical phenotypes of mouse models for OCRD described above, cognitive alterations observed in OCD patients have recently been examined in *Dlgap3* KO mice. Impaired cognitive flexibility was observed in *Dlgap3* KO mice in the context of Pavlovian and instrumental reversal learning (Benzina et al. 2021; Manning et al. 2019; van den Boom et al. 2019), but again with high variability in performance in the latter, considered in parallel with the heterogeneity and inconsistency in the results of reversal learning in OCD patients (Benzina et al. 2021; Chamberlain et al. 2008; Gottwald et al. 2018; Remijnse et al. 2006; Valerius et al. 2008). Altered habit formation was observed, but with inconsistency in terms of sensitivity to reward devaluation (Ehmer et al. 2020; Hadjas et al. 2019) in parallel with inconsistency in the results in human patients (de Wit et al. 2018; Gillan et al. 2014). Cross-species behavioral tasks consistently revealed enhanced negative and impaired positive valence processing in both human patients and *Dlgap3* KO mice (Kajs et al. 2022). Contrary to the early concept of these cognitive alterations as a cause of compulsive behavior, many of these studies have shown results suggesting independence between observed cognitive alterations and compulsive behavior in *Dlgap3* KO mice (Kajs et al. 2022; Benzina et al. 2021; Ehmer et al. 2020; Manning et al. 2019; van den Boom et al. 2019), and one study suggested different circuit mechanisms for cognitive inflexibility and compulsive behavior (Yang et al. 2021). In this context, performance of *Dlgap1* KO mice in these cognitive tasks described above should also be investigated in future studies, in order to strengthen the translational relevance of the animal model.

Concerning the heterogeneity in self-grooming and cognitive performance of mutant mice mentioned above, in general, recent behavioral studies have pointed out similar within-genotype heterogeneity (Freund et al. 2013), with discussion of a variety of possible underlying mechanisms, including genetic drift (Neuro-BSIK Mouse Phenomics Consortium. 2015), epigenetic variation (Talens et al. 2012), maternal, nutritional and social factors (Lathe 2004) and uncontrolled environmental differences (Geuther et al. 2021). Although few clues to the mechanisms underlying the heterogeneity among *Dlgap1* and *3* KO mice have been obtained, except for the results of pedigree analysis being in disagreement with genetic drift and other mechanisms shared among littermates, such as epigenetic factors and parental behavior (Manning et al. 2019), their understanding might reveal a source of clinical heterogeneity of OCRD and is thus important.

From the point of view of mechanism, the roles of DLGAP3 in the development of OCD-like phenotype have been extensively investigated by numerous studies. Deletion in *Dlgap3* has been shown to result in altered synaptic strength and circuit dynamics in the striatum (Burguière et al. 2013; Corbit et al. 2019; Hadjas et al. 2020; Ramírez-Armenta et al. 2022; Welch et al. 2007), especially imbalance between the dynamics of striatal subcircuits due to altered expression of metabotropic glutamate receptor 5 in cortico-striatal synapses (Ade et al. 2016; Glorie et al. 2020; Wan et al. 2011). In contrast, thus far, little has been elucidated about synaptic and circuit functions in *Dlgap1* KO mice. As we speculate on the role of DLGAP1 in synaptic and circuit functions, differences in tissue localization of DLGAP1 and 3 (Kindler et al. 2004; Welch et al. 2004) need to be taken into account. Large-scale quantitative measurement of RNA expression in mouse brain tissues revealed higher expression of *Dlgap1* than of other isoforms in the cerebral cortex and hippocampus, and higher expression of *Dlgap3* than of other isoforms in the striatum (Sjöstedt et al. 2020). Similarities and differences between their expression patterns were observed at the single-cell level in adolescent mice (Zeisel et al. 2018). It should also be considered that the same level of expression of the two molecules in a neuronal population does not necessarily result in the same functionality because of possible differences in their downstream signaling, although such downstream signaling pathways for DLGAPs have not been comparatively studied. It is noteworthy, however, that both DLGAP1 and 3 were proven to interact with SHANK3 (Naisbitt et al. 1999; Zeng et al. 2016), whose (conditional) KO mice exhibited excessive grooming and decreased rearings (Bey et al. 2018; Peça et al. 2011).

Taking all these results into account, two hypotheses can be proposed about the roles of DLGAP1 and 3 in the pathogenesis of OCD-like behavior. First, it can be hypothesized on the basis of protein homology and striatal co-expression that their roles in corticostriatal synapses are complementary to each other, and dysfunction of either one of these leads to OCD-like behavior. Although the gross expression level of DLGAP1 in the striatum is much lower than that of DLGAP3, their expression may be comparable in the subset of cells responsible for the development of compulsive behavioral patterns (Zeisel et al. 2018). This hypothesis must be tested against another hypothesis that DLGAP1 and 3 play roles in different brain areas. A larger impact of deletion in *Dlgap1* in the cerebral cortex and hippocampus is expected from its expression level. Neuronal populations constituting the basolateral amygdala are known to be very similar to those in the cerebral cortex in different aspects except for laminar organization (see McDonald (2020) and references cited therein for detailed description of this similarity: e.g., the presence of 70–80% of glutamatergic pyramidal neurons and mainly GABAergic subpopulations of interneurons identified with a similar set of calcium-binding proteins as markers that are interconnected with similar synaptic organization and have similar intrinsic electrophysiological properties), and it is therefore suggested that deletion of *Dlgap1* might also impact the basolateral amygdala as well. Although most animal models of OCRD exhibit anatomical and functional defects in cortico-striatal synapses (Nagarajan et al. 2018; Shmelkov et al. 2010; Welch et al. 2007), the marked increase in transmission at thalamo-amygdala and cortico-striatal synapses observed in *Spred2* KO mice (Ullrich et al. 2018) suggested that synaptic reorganizations in different brain areas could underlie OCD-like phenotypes, including those involving the amygdala.

With these plausible hypotheses in mind, despite the scarcity of evidence about functional roles of DLGAP1, one can draw an important translational implication useful for directing future research from the fact that mutations in both of the genes for the two highly homological proteins mediating synaptic plasticity in overlapping, but different neuronal populations result in OCD-like phenotypes: Mutations in the two genes and associated synaptic dysfunction in different brain regions may serve as two different axes that span a broader part of the OCRD spectrum than do mutations in *Dlgap3* alone. In fact, a sex difference in scratching behavior of our KO mice was observed in our study, but not in self-grooming of *Dlgap3* KO mice in previous studies, which would not be explained entirely by methodological differences between the studies (see our Fig. 5d–f and Fig. S9 of Manning et al. (2021) for comparison). In this context, whether our female mutant mice exhibit pronounced pre-pulse inhibition as observed in female OCD patients (Ahmari et al. 2016; Steinman et al. 2016) is an important question to be addressed in future studies. While translational relevance of *Dlgap*3 has been shown by cross-species behavioral tests in both human patients and animal models (Benzina et al. 2021; Kajs et al. 2022), such a comparison is sometimes confronted with heterogeneity of human OCRD patients (Benzina et al. 2021). Although previous studies have discussed this heterogeneity in parallel with the within-genotype heterogeneity as mentioned above, human phenotypes are obviously subjected to large genetic variability as well. Additional use of *Dlgap*1 KO mice in this line of study may therefore advance the characterization of the disease spectrum. Apart from the promising aspects of the translational studies described above, we point out the discrepancy in terms of the effectiveness of treatment with SSRI between human patients and animal models. The responsiveness of human OCD patients to antidepressants is reported to be between 40 and 60% (Del Casale et al. 2019; Soomro et al. 2008). The responsiveness of trichotillomania patients to antidepressants is controversial (Johnson and El-Alfy 2016; Lochner et al. 2005; McGuire et al. 2014). The observed responsiveness to antidepressants shared among mutant animal models including ours is therefore not sufficient to account for the diversity of OCRD. This gap should be approached by further analysis of the phenome of human OCRD patients (Wu et al. 2022) and its association with genomic variation (Jain et al. 2023), together with less-frequently studied animal models with compulsive behavior (Chou-Green et al. 2003; Delgado-Acevedo et al. 2019; Hill et al. 2007), especially, those with mutations in genes involved in pathways bridging pathophysiology of OCD-like phenotypes and serotonergic systems (Chou-Green et al. 2003).

## Conclusion

This study showed that genetic deletion of *Dlgap1* in mice caused late development of OCD-like phenotypes including skin lesions, excessive self-grooming and anxiety-like behavior, which was responsive to treatment with fluvoxamine. A significant sex difference was observed in pronounced scratching behavior characterizing excessive self-grooming of the mutant mice. The protein homology and differences in tissue localization of DLGAP1 and 3 together with similar OCD-like phenotypes exhibited by their KO mice suggest that further studies of *Dlgap1* KO mice, in comparison with *Dlgap3* KO mice, should be performed to account for a broader part of the phenotypes of the OCRD spectrum, to clarify its underlying neuronal mechanisms.

## Electronic supplementary material

Below is the link to the electronic supplementary material.

Supplementary Material 1

Supplementary Material 2

## Declarations

### Author Statement

All animal experiments complied with the ARRIVE guidelines and were approved by the Institutional Animal Care and Use Committee of the School of Medicine, Nihon University (Approval number: AP20MED064 and 067, AP22MED042 and 061, AP23MED015).

The work has not been published, nor has it been submitted elsewhere. All the authors have approved the current version of the manuscript.
