# Supplementary material for: Late development of OCD-like phenotypes in Dlgap1 knockout mice
Source: Psychopharmacology (Berl). 2024 Aug 23;242(1):215–31. doi: 10.1007/s00213-024-06668-9 (PMC11742909; doi:10.1007/s00213-024-06668-9)
Supplement: Supplementary file 1 — Supplementary Material 1 [file 213_2024_6668_MOESM1_ESM.pdf]

**Suppl. Table 1** Results of modified-SHIRPA

| | | Female | | | Male | | |
|---|---|---|---|---|---|---|---|
| | | WT | HT | KO | WT | HT | KO |
| Tests in viewing jar | Coat color | 0.00 ± 0.00 | 0.00 ± 0.00 | 0.00 ± 0.00 | 0.00 ± 0.00 | 0.00 ± 0.00 | 0.00 ± 0.00 |
| | Hair length | 0.00 ± 0.00 | 0.00 ± 0.00 | 0.00 ± 0.00 | 0.00 ± 0.00 | 0.00 ± 0.00 | 0.00 ± 0.00 |
| | Hair morphology | 0.00 ± 0.00 | 0.00 ± 0.00 | 0.00 ± 0.00 | 0.00 ± 0.00 | 0.00 ± 0.00 | 0.00 ± 0.00 |
| | Respiratory rate | 2.00 ± 0.00 | 2.00 ± 0.00 | 2.00 ± 0.00 | 2.00 ± 0.00 | 2.00 ± 0.00 | 2.00 ± 0.00 |
| | Tremor | 0.00 ± 0.00 | 0.00 ± 0.00 | 0.00 ± 0.00 | 0.00 ± 0.00 | 0.00 ± 0.00 | 0.00 ± 0.00 |
| | Body position | 3.58 ± 0.19 | 3.58 ± 0.23 | 3.58 ± 0.19 | 3.58 ± 0.19 | 3.58 ± 0.19 | 4.17 ± 0.21 |
| | Spontaneous activity | 1.34 ± 0.14 | 1.25 ± 0.13 | 1.75 ± 0.25 | 1.67 ± 0.11 | 1.34 ± 0.14 | 2.08 ± 0.19 |
| | Defecation (number of feces) | 1.92 ± 0.51 | 4.75 ± 1.09$^{\#\#\#}$ | 5.5 ± 0.77$^{***}$ | 3.25 ± 0.90 | 5.75 ± 0.74$^{\#\#\#}$ | 7.33 ± 0.97$^{***}$ |
| | Urination | 0.08 ± 0.08 | 0.50 ± 0.15 | 0.42 ± 0.15$^{*}$ | 0.33 ± 0.14 | 0.58 ± 0.15 | 0.58 ± 0.15$^{*}$ |
| | Grooming time (seconds) | 18.51 ± 6.16 | 18.00 ± 10.38 | 14.26 ± 9.34 | 17.11 ± 9.18 | 18.72 ± 6.84 | 16.95 ± 7.98 |
| | Number of grooming bouts | 8.17 ± 4.63 | 6.75 ± 3.60 | 6.50 ± 3.18 | 7.08 ± 4.85 | 9.08 ± 5.47 | 7.00 ± 4.61 |
| Tests in arena | Time elapsed before the mouse starts to move (seconds) | 4.19 ± 1.9 | 3.19 ± 0.9 | 3.31 ± 1.3 | 4.77 ± 1.6 | 1.67 ± 0.4 | 2.06 ± 0.5 |
| | Locomotor activity (number of squares) | 16.33 ± 2.9 | 14.50 ± 2.2 | 17.17 ± 2.3 | 17.0 ± 1.9 | 19.25 ± 2.2 | 18.92 ± 1.4 |
| | Transfer arousal | 2.08 ± 0.19 | 2.17 ± 0.11 | 2.25 ± 0.18 | 2.67 ± 0.19 | 2.42 ± 0.15 | 2.42 ± 0.15 |
| | Piloerection | 0.00 ± 0.00 | 0.00 ± 0.00 | 0.00 ± 0.00 | 0.00 ± 0.00 | 0.00 ± 0.00 | 0.00 ± 0.00 |
| | Startle response | 1.00 ± 0.00 | 1.00 ± 0.00 | 1.00 ± 0.00 | 1.00 ± 0.00 | 1.00 ± 0.00 | 1.00 ± 0.00 |
| | Gait | 0.00 ± 0.00 | 0.00 ± 0.00 | 0.25 ± 0.18 | 0.00 ± 0.00 | 0.00 ± 0.00 | 0.00 ± 0.00 |
| | Pelvic elevation | 2.25 ± 0.13 | 2.17 ± 0.11 | 2.17 ± 0.17 | 2.00 ± 0.00 | 2.08 ± 0.08 | 2.08 ± 0.08 |
| | Tail elevation | 1.17 ± 0.11 | 1.33 ± 0.14 | 1.75 ± 0.13$^{**}$ | 1.42 ± 0.15 | 1.58 ± 0.15 | 1.83 ± 0.11$^{**}$ |
| | Touch escape | 2.83 ± 0.11 | 2.50 ± 0.15 | 2.33 ± 0.14$^{**}$ | 2.83 ± 0.11 | 2.67 ± 0.14 | 2.33 ± 0.14$^{**}$ |
| Tests above arena/on arena | Visual placing | 2.00 ± 0.00 | 2.00 ± 0.00 | 2.00 ± 0.00 | 2.00 ± 0.00 | 2.00 ± 0.00 | 2.00 ± 0.00 |
| | Grip strength | 2.33 ± 0.14 | 2.08 ± 0.08 | 2.00 ± 0.12 | 2.08 ± 0.08 | 2.00 ± 0.00 | 2.00 ± 0.00 |
| | Body tone | 1.00 ± 0.00 | 1.00 ± 0.00 | 1.00 ± 0.00 | 1.00 ± 0.00 | 1.00 ± 0.00 | 1.00 ± 0.00 |
| | Head morphology | 0.00 ± 0.00 | 0.00 ± 0.00 | 0.00 ± 0.00 | 0.00 ± 0.00 | 0.00 ± 0.00 | 0.00 ± 0.00 |
| | Pinna reflex | 1.00 ± 0.00 | 1.00 ± 0.00 | 1.00 ± 0.00 | 1.00 ± 0.00 | 1.00 ± 0.00 | 1.00 ± 0.00 |
| | Pinna morphology | 0.00 ± 0.00 | 0.00 ± 0.00 | 0.00 ± 0.00 | 0.00 ± 0.00 | 0.00 ± 0.00 | 0.00 ± 0.00 |
| | Corneal reflex | 1.00 ± 0.00 | 1.00 ± 0.00 | 1.00 ± 0.00 | 1.00 ± 0.00 | 1.00 ± 0.00 | 1.00 ± 0.00 |
| | Toe pinch | 3.00 ± 0.00 | 3.00 ± 0.00 | 3.00 ± 0.00 | 3.00 ± 0.00 | 3.00 ± 0.00 | 3.00 ± 0.00 |
| | Body length (mm) | 88.75 ± 0.76$^{\dagger\dagger\dagger}$ | 87.33 ± 1.33$^{\dagger\dagger\dagger}$ | 86.58 ± 1.02$^{\dagger\dagger\dagger}$ | 93.67 ± 0.86 | 94.42 ± 1.08 | 90.25 ± 1.52 |
| | Tail length (mm) | 70.17 ± 0.63$^{\dagger\dagger\dagger}$ | 68.92 ± 2.81$^{\dagger\dagger\dagger}$ | 68.17 ± 0.69$^{\dagger\dagger\dagger}$ | 72.75 ± 0.70 | 72.00 ± 0.66 | 71.00 ± 0.98 |
| | Body weight (g) | 19.14 ± 1.25$^{\dagger\dagger\dagger}$ | 18.74 ± 1.30$^{\#,\dagger\dagger\dagger}$ | 19.35 ± 1.90$^{\dagger\dagger\dagger}$ | 24.35 ± 1.78 | 22.42 ± 1.85$^{\#}$ | 22.93 ± 2.07 |
| | BMI | 2.43 ± 0.06$^{\dagger\dagger}$ | 2.46 ± 0.09$^{\dagger\dagger}$ | 2.59 ± 0.08$^{\ddagger,\dagger\dagger}$ | 2.77 ± 0.03 | 2.52 ± 0.06 | 2.87 ± 0.13$^{\ddagger}$ |
| | Tail morphology | 0.00 ± 0.00 | 0.00 ± 0.00 | 0.00 ± 0.00 | 0.00 ± 0.00 | 0.00 ± 0.00 | 0.00 ± 0.00 |
| | Lacrimation | 0.00 ± 0.00 | 0.00 ± 0.00 | 0.00 ± 0.00 | 0.00 ± 0.00 | 0.00 ± 0.00 | 0.00 ± 0.00 |
| | Palpebral closure | 0.00 ± 0.00 | 0.00 ± 0.00 | 0.00 ± 0.00 | 0.00 ± 0.00 | 0.00 ± 0.00 | 0.00 ± 0.00 |
| | Cornea | 0.00 ± 0.00 | 0.00 ± 0.00 | 0.00 ± 0.00 | 0.00 ± 0.00 | 0.00 ± 0.00 | 0.00 ± 0.00 |
| | Pupil | 0.00 ± 0.00 | 0.00 ± 0.00 | 0.00 ± 0.00 | 0.00 ± 0.00 | 0.00 ± 0.00 | 0.00 ± 0.00 |
| | Eye size/color/shape | 0.00 ± 0.00 | 0.00 ± 0.00 | 0.00 ± 0.00 | 0.00 ± 0.00 | 0.00 ± 0.00 | 0.00 ± 0.00 |
| | Whisker morphology | 0.00 ± 0.00 | 0.00 ± 0.00 | 0.00 ± 0.00 | 0.00 ± 0.00 | 0.00 ± 0.00 | 0.00 ± 0.00 |
| | Tooth morphology | 0.00 ± 0.00 | 0.00 ± 0.00 | 0.00 ± 0.00 | 0.00 ± 0.00 | 0.00 ± 0.00 | 0.00 ± 0.00 |
| | Provoked biting | 1.00 ± 0.00 | 1.00 ± 0.00 | 1.00 ± 0.00 | 1.00 ± 0.00 | 1.00 ± 0.00 | 1.00 ± 0.00 |
| | Salivation | 1.00 ± 0.00 | 1.00 ± 0.00 | 1.00 ± 0.00 | 1.00 ± 0.00 | 1.00 ± 0.00 | 1.00 ± 0.00 |
| | Heart rate | 0.00 ± 0.00 | 0.00 ± 0.00 | 0.00 ± 0.00 | 0.00 ± 0.00 | 0.00 ± 0.00 | 0.00 ± 0.00 |
| | Abdominal tone | 0.00 ± 0.00 | 0.00 ± 0.00 | 0.00 ± 0.00 | 0.00 ± 0.00 | 0.00 ± 0.00 | 0.00 ± 0.00 |
| | Skin color | 1.00 ± 0.00 | 1.00 ± 0.00 | 1.00 ± 0.00 | 1.00 ± 0.00 | 1.00 ± 0.00 | 1.00 ± 0.00 |
| | Limb morphology | 0.00 ± 0.00 | 0.00 ± 0.00 | 0.00 ± 0.00 | 0.00 ± 0.00 | 0.00 ± 0.00 | 0.00 ± 0.00 |
| | Limb tone | 1.00 ± 0.00 | 1.00 ± 0.00 | 1.00 ± 0.00 | 1.00 ± 0.00 | 1.00 ± 0.00 | 1.00 ± 0.00 |
| | Wire maneuver | 0.17 ± 0.11 | 0.25 ± 0.13 | 0.25 ± 0.13 | 0.42 ± 0.19 | 0.33 ± 0.14 | 0.42 ± 0.19 |
| | Righting reflex | 0.00 ± 0.00 | 0.00 ± 0.00 | 0.00 ± 0.00 | 0.00 ± 0.00 | 0.00 ± 0.00 | 0.00 ± 0.00 |
| | Contact righting reflex | 1.00 ± 0.00 | 1.00 ± 0.00 | 1.00 ± 0.00 | 1.00 ± 0.00 | 1.00 ± 0.00 | 1.00 ± 0.00 |
| | Negative geotaxis | 0.00 ± 0.00 | 0.00 ± 0.00 | 0.00 ± 0.00 | 0.00 ± 0.00 | 0.00 ± 0.00 | 0.00 ± 0.00 |
| Additional | Fear | 0.00 ± 0.00 | 0.00 ± 0.00 | 0.00 ± 0.00 | 0.00 ± 0.00 | 0.00 ± 0.00 | 0.00 ± 0.00 |
| | Irritability | 0.00 ± 0.00 | 0.08 ± 0.08 | 0.08 ± 0.08 | 0.00 ± 0.00 | 0.08 ± 0.08 | 0.00 ± 0.00 |
| | Aggression | 0.25 ± 0.13 | 0.00 ± 0.00$^{\$}$ | 0.08 ± 0.08 | 0.17 ± 0.11 | 0.42 ± 0.15 | 0.17 ± 0.11 |
| | Vocalization | 0.00 ± 0.00 | 0.08 ± 0.08 | 0.17 ± 0.11 | 0.00 ± 0.00 | 0.08 ± 0.08 | 0.17 ± 0.11 |

Mean and its standard error of the indicated morphological and behavioral parameters for each combination of sex and genotype are shown in the table. The data for each item were analyzed by performing mixed (ART-)ANOVA (using genotype and sex as within- and between-subject factors, respectively) followed by post-hoc tests with Bonferroni corrections. Only statistically significant differences confirmed by post-hoc tests are shown by the following symbols: KO vs WT, $^{***}$ $p < 0.001$, $^{**}$ $p < 0.01$, $^{*}$ $p < 0.05$; HT vs WT, $^{\#\#\#}$ $p < 0.001$, $^{\#}$ $p < 0.05$; KO vs HT, $^{\ddagger}$ $p < 0.05$; female vs male, $^{\dagger\dagger\dagger}$ $p < 0.001$, $^{\dagger\dagger}$ $p < 0.01$, $^{\dagger}$ $p < 0.05$; female HT vs male HT, $^{\$}$ $p < 0.05$.

**Suppl. Table 2.** Two-way mixed (ART-)ANOVA of results of open-field test using sex and genotype as factors.

| | Two-way mixed (ART-)ANOVA | | | | post-hoc test | |
|---|---|---|---|---|---|---|
| | Factor | DFn, DFd | *F* | *p*-value | | (adjusted) *p*-value |
| † Locomotor activity | Genotype | 2, 36 | 67.27 | < 0.001 | WT vs KO | < 0.001 |
| | Sex | 1, 18 | 0.08 | 0.778 | WT vs HT | 1.000 |
| | Genotype × sex | 2, 36 | 0.31 | 0.735 | HT vs KO | < 0.001 |
| † Time spent in center | Genotype | 2, 36 | 48.72 | < 0.001 | WT vs KO | < 0.001 |
| | Sex | 1, 18 | 0.81 | 0.381 | WT vs HT | 0.008 |
| | Genotype × sex | 2, 36 | 0.18 | 0.834 | HT vs KO | < 0.001 |
| † Time spent in periphery | Genotype | 2, 36 | 18.01 | < 0.001 | WT vs KO | < 0.001 |
| | Sex | 1, 18 | 0.34 | 0.565 | WT vs HT | 0.098 |
| | Genotype × sex | 2, 36 | 0.07 | 0.928 | HT vs KO | 0.025 |
| † Rearing | Genotype | 2, 36 | 13.15 | < 0.001 | WT vs KO | < 0.001 |
| | Sex | 1, 18 | 0.97 | 0.337 | WT vs HT | 0.375 |
| | Genotype × sex | 2, 36 | 0.25 | 0.781 | HT vs KO | 0.004 |
| † Number of grooming bouts | Genotype | 2, 36 | 55.19 | < 0.001 | WT vs KO | < 0.001 |
| | Sex | 1, 18 | 1.40 | 0.245 | WT vs HT | 1.000 |
| | Genotype × sex | 2, 36 | 0.86 | 0.440 | HT vs KO | < 0.001 |
| Grooming time | Genotype | 2, 36 | 81.28 | < 0.001 | WT vs KO | < 0.001 |
| | Sex | 1, 18 | 3.32 | 0.035 | WT vs HT | 1.000 |
| | Genotype × sex | 2, 36 | 2.70 | 0.107 | HT vs KO | < 0.001 |
| † Latency to leave center | Genotype | 2, 36 | 1.06 | 0.356 | | |
| | Sex | 1, 18 | 1.14 | 0.300 | | |
| | Genotype × sex | 2, 36 | 0.08 | 0.922 | | |

† Data were analyzed by performing two-way mixed ART-ANOVA.

**Suppl. Table 3.** One-way ANCOVA of non-scratching time in KO mice measured in 24-hour behavioral monitoring using treatment as a factor.

| | Covariate | BIC | Likelihood | Main effect of treatment | Shapiro-Wilk test | Levene test |
|---|---|---|---|---|---|---|
| Model-0 | (0) | 376.19 | -182.10 | $F_{1, 17}$ = 6.94 $p$ = 0.017 | 0.080 | 0.261 |
| Model-1 | (0), (1) | 378.36 | -181.69 | $F_{1, 16}$ = 6.70 $p$ = 0.020 | 0.044 | 0.269 |
| Model-2 | (0), (2) | 371.05 | -176.54 | $F_{1, 15}$ = 10.54 $p$ = 0.002 | 0.115 | 0.027 |
| Model-3 | (0), (3) | 377.19 | -179.61 | $F_{1, 15}$ = 5.82 $p$ = 0.029 | 0.173 | 0.049 |
| Model-4 | (0), (4) | 369.97 | -176.00 | $F_{1, 15}$ = 9.16 $p$ = 0.008 | 0.437 | 0.013 |
| Model-5 | (0), (1), (2) | 369.78 | -174.41 | $F_{1, 14}$ = 12.28 $p$ = 0.004 | 0.005 | 0.219 |
| Model-6 | (0), (1), (3) | 379.53 | -179.28 | $F_{1, 14}$ = 5.68 $p$ = 0.032 | 0.079 | 0.177 |
| Model-7 | (0), (1), (4) | 371.90 | -175.47 | $F_{1, 14}$ = 9.27 $p$ = 0.009 | 0.184 | 0.067 |
| Model-8 | (0), (2), (4) | 370.72 | -173.38 | $F_{1, 13}$ = 12.39 $p$ = 0.004 | 0.464 | 0.035 |
| Model-9 | (0), (3), (4) | 371.85 | -173.94 | $F_{1, 13}$ = 11.37 $p$ = 0.005 | 0.858 | 0.001 |
| Model-10 | (0), (1), (2), (4) | 369.03 | -171.03 | $F_{1, 12}$ = 15.71 $p$ = 0.002 | 0.243 | 0.150 |
| Model-11 | (0), (1), (3), (4) | 373.12 | -173.08 | $F_{1, 12}$ = 11.85 $p$ = 0.005 | 0.960 | 0.002 |

Analysis was performed for models with different sets of covariates chosen from (0) sex, (1) onset age, (2) two baseline grooming levels, (3) three baseline grooming levels and (4) the pair of grooming time and number of grooming bouts. Nonhomogeneity of regression slopes between males and females was detected for only covariate (2), and this covariate was expanded into one with sex-wise levels. Discrete baseline grooming levels represented by covariate (2) and (3) were identified by *k*-means clustering and are shown in Fig. 4(a). Analyses with more grooming levels than four led to consistently worse BIC values and their results have therefore been omitted. Results of Shapiro-Wilk tests and Levene tests for residuals of regression are also shown. Levene test was performed across both sexes and treatment groups.

**Suppl. Table 4.** Two-way ANCOVA of number of non-scratching bouts in KO mice counted in 24-hour behavioral monitoring using sex and treatment as factors.

| | Covariate | BIC | Likelihood | Main effect of treatment | Main effect of sex | Interaction | Shapiro-Wilk test | Levene test |
|---|---|---|---|---|---|---|---|---|
| Model-0 | – | 181.47 | -83.25 | $F_{1, 16}$ = 25.06<br>$p$ < 0.001 | $F_{1, 16}$ = 7.37<br>$p$ = 0.015 | $F_{1, 16}$ = 3.58<br>$p$ = 0.077 | 0.023 | 0.944 |
| Model-1 | (1) | 181.31 | -81.67 | $F_{1, 15}$ = 27.13<br>$p$ < 0.001 | $F_{1, 15}$ = 8.47<br>$p$ = 0.011 | $F_{1, 15}$ = 4.19<br>$p$ = 0.059 | 0.759 | 0.902 |
| Model-2 | (2) | 184.36 | -83.19 | $F_{1, 15}$ = 23.29<br>$p$ < 0.001 | $F_{1, 15}$ = 6.83<br>$p$ = 0.020 | $F_{1, 15}$ = 3.45<br>$p$ = 0.083 | 0.722 | 0.628 |
| Model-3 | (3) | 187.35 | -83.19 | $F_{1, 14}$ = 20.49<br>$p$ < 0.001 | $F_{1, 14}$ = 5.91<br>$p$ = 0.029 | $F_{1, 14}$ = 3.17<br>$p$ = 0.097 | 0.721 | 0.631 |
| Model-4 | (4) | 184.60 | -81.82 | $F_{1, 14}$ = 20.51<br>$p$ < 0.001 | $F_{1, 14}$ = 3.60<br>$p$ = 0.079 | $F_{1, 14}$ = 4.00<br>$p$ = 0.065 | 0.208 | 0.950 |
| Model-5 | (1), (2) | 184.21 | -81.62 | $F_{1, 14}$ = 25.13<br>$p$ < 0.001 | $F_{1, 14}$ = 7.83<br>$p$ = 0.014 | $F_{1, 14}$ = 3.99<br>$p$ = 0.066 | 0.628 | 0.905 |
| Model-6 | (1), (3) | 187.06 | -81.55 | $F_{1, 13}$ = 22.72<br>$p$ = 0.02 | $F_{1, 13}$ = 7.08<br>$p$ = 0.020 | $F_{1, 13}$ = 3.83<br>$p$ = 0.072 | 0.814 | 0.917 |
| Model-7 | (1), (4) | 185.50 | -80.77 | $F_{1, 13}$ = 21.66<br>$p$ < 0.001 | $F_{1, 13}$ = 4.41<br>$p$ = 0.056 | $F_{1, 13}$ = 4.17<br>$p$ = 0.062 | 0.657 | 0.898 |
| Model-8 | (2), (4) | 186.94 | -81.49 | $F_{1, 13}$ = 17.66<br>$p$ = 0.001 | $F_{1, 13}$ = 4.02<br>$p$ = 0.066 | $F_{1, 13}$ = 3.33<br>$p$ = 0.091 | 0.239 | 0.913 |
| Model-9 | (3), (4) | 178.78 | -75.91 | $F_{1, 12}$ = 26.89<br>$p$ < 0.001 | $F_{1, 12}$ = 3.54<br>$p$ = 0.084 | $F_{1, 12}$ = 9.92<br>$p$ = 0.008 | 0.772 | 0.242 |
| Model-10 | (1), (2), (4) | 187.92 | -80.48 | $F_{1, 12}$ = 18.54<br>$p$ = 0.001 | $F_{1, 12}$ = 4.72<br>$p$ = 0.050 | $F_{1, 12}$ = 3.48<br>$p$ = 0.087 | 0.524 | 0.918 |
| Model-11 | (1), (3), (4) | 178.74 | -74.39 | $F_{1, 11}$ = 29.50<br>$p$ < 0.001 | $F_{1, 11}$ = 4.55<br>$p$ = 0.056 | $F_{1, 11}$ = 10.64<br>$p$ = 0.008 | 0.882 | 0.643 |

Analysis was performed for models with different sets of covariates chosen from (1) onset age, (2) two baseline grooming levels, (3) three baseline grooming levels and (4) the pair of grooming time and number of grooming bouts. Discrete baseline grooming levels represented by covariate (2) and (3) were identified by *k*-means clustering and are shown in Fig. 4(a). Analyses with more grooming levels than four led to consistently worse BIC values and their results have therefore been omitted. Results of Shapiro-Wilk tests and Levene tests for residuals of regression are also shown. For the optimal model, model-11, post-hoc pairwise tests yielded the following $p$-values: fluvoxamine-treated female vs vehicle-treated female, $p$ = 0.021; fluvoxamine-treated male vs vehicle-treated male, $p$ = 0.526; fluvoxamine-treated female vs fluvoxamine-treated male, $p$ = 0.977; vehicle-treated female vs vehicle-treated male, $p$ = 0.022.

**Suppl. Table 5.** One-way ANCOVA of number of scratching bouts in KO mice counted in 24-hour behavioral monitoring using treatment as a factor.

| | Covariate | BIC | Likelihood | Main effect of treatment | Shapiro-Wilk test | Levene test |
|---|---|---|---|---|---|---|
| Model-0 | (0) | 288.90 | -138.46 | $F_{1,\,17} = 44.95$ $p < 0.001$ | 0.047 | 0.109 |
| Model-1 | (0), (1) | 290.30 | -137.66 | $F_{1,\,16} = 45.42$ $p < 0.001$ | 0.054 | 0.118 |
| Model-2 | (0), (2) | 283.60 | -132.81 | $F_{1,\,15} = 69.42$ $p < 0.001$ | 0.010 | 0.330 |
| Model-3 | (0), (3) | 289.38 | -135.78 | $F_{1,\,15} = 43.75$ $p < 0.001$ | 0.349 | 0.613 |
| Model-4 | (0), (4) | 291.62 | -136.82 | $F_{1,\,15} = 39.81$ $p < 0.001$ | 0.728 | 0.126 |
| Model-5 | (0), (1), (2) | 278.85 | -128.94 | $F_{1,\,14} = 95.83$ $p < 0.001$ | 0.052 | 0.540 |
| Model-6 | (0), (1), (3) | 291.22 | -135.12 | $F_{1,\,14} = 43.52$ $p < 0.001$ | 0.676 | 0.699 |
| Model-7 | (0), (1), (4) | 293.49 | -136.26 | $F_{1,\,14} = 39.79$ $p < 0.001$ | 0.677 | 0.140 |
| Model-8 | (0), (2), (4) | 284.97 | -130.50 | $F_{1,\,13} = 63.14$ $p < 0.001$ | 0.004 | 0.322 |
| Model-9 | (0), (3), (4) | 295.33 | -135.68 | $F_{1,\,13} = 35.57$ $p < 0.001$ | 0.248 | 0.614 |
| Model-10 | (0), (1), (2), (4) | 276.77 | -124.90 | $F_{1,\,12} = 107.19$ $p < 0.001$ | 0.180 | 0.606 |
| Model-11 | (0), (1), (3), (4) | 297.16 | -135.10 | $F_{1,\,12} = 35.30$ $p < 0.001$ | 0.546 | 0.753 |

Analysis was performed for models with different sets of covariates chosen from (0) sex, (1) onset age, (2) two baseline grooming levels, (3) three baseline grooming levels and (4) the pair of grooming time and number of grooming bouts. Nonhomogeneity of regression slopes between males and females was detected for only covariate (2), and this covariate was expanded into one with sex-wise levels. Discrete baseline grooming levels represented by covariate (2) and (3) were identified by *k*-means clustering and are shown in Fig. 4(a). Analyses with more grooming levels than four led to consistently worse BIC values and their results have therefore been omitted. Results of Shapiro-Wilk tests and Levene tests for residuals of regression are also shown. Levene test was performed across both sexes and treatment groups.

**Suppl. Table 6.** Two-way ART-ANOVA of change in skin-lesion scores between before and after treatment using sex and treatment as factors.

| | | Δ skin-lesion score |
|---|---|---|
| ART-ANOVA | Sex | $F_{1,8} = 4.08 \times 10^{-41}$<br>$p = 1.000$ |
| | Treatment | $F_{1,8} = 200.0$<br>$p < 0.001$ |
| | Sex × treatment | $F_{1,8} = 0.00$<br>$p = 1.000$ |
| post-hoc tests | *p*-value in Wilcoxon rank-sum test comparing treatment groups | < 0.001 |

**Suppl. Table 7.** Two-way mixed ART-ANOVA of non-scratching time and numbers of scratching and non-scratching bouts of vehicle-treated mice in 24-hour behavioral monitoring using sex and genotype as factors.

| | | Non-scratching time | Number of non-scratching bouts | Number of scratching bouts |
|---|---|---|---|---|
| ART-ANOVA | Sex | $F_{1, 8} = 8.70$<br>$p = 0.018$ | $F_{1, 8} = 14.34$<br>$p = 0.005$ | $F_{1, 8} = 37.95$<br>$p < 0.001$ |
| | Genotype | $F_{2, 16} = 13.27$<br>$p < 0.001$ | $F_{2, 16} = 24.10$<br>$p < 0.001$ | $F_{2, 16} = 41.76$<br>$p < 0.001$ |
| | Sex × genotype | $F_{2, 16} = 1.69$<br>$p = 0.216$ | $F_{2, 16} = 1.45$<br>$p = 0.265$ | $F_{2, 16} = 16.79$<br>$p < 0.001$ |
| post-hoc tests | female vs male<br>Wilcoxon rank-sum test<br>*p*-value<br>with Bonferroni corrections | 0.285 | 0.042 | KO vs HT 0.048<br>KO vs WT 0.024<br>HT vs WT 0.930 |
| | Main effect of genotype in<br>Friedman rank-sum test<br>*p*-value | < 0.001 | < 0.001 | female 0.007<br>male 0.022 |
| | *p*-value in pairwise Wilcoxon<br>signed rank test with<br>Bonferroni corrections | KO vs HT 0.006<br>KO vs WT 0.006<br>HT vs WT 1.000 | KO vs HT 0.006<br>KO vs WT 0.006<br>HT vs WT 1.000 | female<br>KO vs HT 0.188<br>KO vs WT 0.188<br>HT vs WT 0.188<br>male<br>KO vs HT 0.188<br>KO vs WT 0.188<br>HT vs WT 1.000 |

**Suppl. Table 8.** The same analyses as for Suppl. Tables 3 and 4, with different thresholds for detecting non-scratching bouts (TD) and different cutoffs for concatenating separate non-scratching bouts with short intervals (CC) in four 30-minute segments excerpted from the 24-hour behavioral monitoring.

| | | Number of non-scratching bouts | | | | | |
|---|---|---|---|---|---|---|---|
| | | TD-3/CC-6 | TD-1/CC-6 | TD-3/CC-3 | TD-1/CC-3 | TD-3/CC-1 | TD-1/CC-1 |
| | Optimal set of covariates | Number of grooming bouts, grooming time, three baseline levels | Onset age, number of grooming bouts, grooming time, three baseline levels | Number of grooming bouts, grooming time, three baseline levels | Onset age, number of grooming bouts, grooming time, three baseline levels | Number of grooming bouts, grooming time, three baseline levels | Onset age, number of grooming bouts, grooming time, three baseline levels |
| ANCOVA | Sex | $F_{1, 12} = 0.66$<br>$p = 0.432$ | $F_{1, 11} = 0.90$<br>$p = 0.363$ | $F_{1, 12} = 1.02$<br>$p = 0.332$ | $F_{1, 12} = 1.67$<br>$p = 0.221$ | $F_{1, 12} = 1.64$<br>$p = 0.224$ | $F_{1, 12} = 2.23$<br>$p = 0.161$ |
| | Treatment | $F_{1, 12} = 31.75$<br>$p < 0.001$ | $F_{1, 11} = 29.84$<br>$p < 0.001$ | $F_{1, 12} = 38.21$<br>$p < 0.001$ | $F_{1, 12} = 33.03$<br>$p < 0.001$ | $F_{1, 12} = 42.88$<br>$p < 0.001$ | $F_{1, 12} = 36.00$<br>$p < 0.001$ |
| | Sex × treatment | $F_{1, 12} = 6.14$<br>$p = 0.029$ | $F_{1, 11} = 5.41$<br>$p = 0.040$ | $F_{1, 12} = 6.34$<br>$p = 0.027$ | $F_{1, 12} = 5.11$<br>$p = 0.043$ | $F_{1, 12} = 7.07$<br>$p = 0.021$ | $F_{1, 12} = 5.56$<br>$p = 0.036$ |
| post-hoc tests | Main effect of treatment<br>*p*-value | female<br>$F_{1, 4} = 156.0$<br>$p < 0.001$<br>male<br>$F_{1, 4} = 4.11$<br>$p = 0.113$ | female<br>$F_{1, 3} = 203.0$<br>$p < 0.001$<br>male<br>$F_{1, 3} = 3.01$<br>$p = 0.181$ | female<br>$F_{1, 4} = 131.0$<br>$p < 0.001$<br>male<br>$F_{1, 4} = 4.59$<br>$p = 0.099$ | female<br>$F_{1, 3} = 132.0$<br>$p = 0.001$<br>male<br>$F_{1, 3} = 2.87$<br>$p = 0.189$ | female<br>$F_{1, 4} = 103.0$<br>$p < 0.001$<br>male<br>$F_{1, 4} = 4.39$<br>$p = 0.104$ | female<br>$F_{1, 3} = 101.5$<br>$p = 0.002$<br>male<br>$F_{1, 3} = 2.65$<br>$p = 0.202$ |
| | Main effect of sex<br>*p*-value | Fluvoxamine<br>$F_{1, 4} = 1.39$<br>$p = 0.304$<br>Vehicle<br>$F_{1, 4} = 6.18$<br>$p = 0.068$ | Fluvoxamine<br>$F_{1, 3} = 0.62$<br>$p = 0.489$<br>Vehicle<br>$F_{1, 3} = 5.54$<br>$p = 0.100$ | Fluvoxamine<br>$F_{1, 4} = 1.15$<br>$p = 0.343$<br>Vehicle<br>$F_{1, 4} = 8.26$<br>$p = 0.045$ | Fluvoxamine<br>$F_{1, 3} = 0.56$<br>$p = 0.510$<br>Vehicle<br>$F_{1, 3} = 6.14$<br>$p = 0.090$ | Fluvoxamine<br>$F_{1, 4} = 1.04$<br>$p = 0.366$<br>Vehicle<br>$F_{1, 4} = 12.10$<br>$p = 0.025$ | Fluvoxamine<br>$F_{1, 3} = 0.52$<br>$p = 0.524$<br>Vehicle<br>$F_{1, 3} = 7.80$<br>$p = 0.068$ |

| | | Non-scratching time | |
|---|---|---|---|
| | | TD-3 | TD-1 |
| | Optimal set of covariates | Onset age, number of grooming bouts, grooming time | Onset age, number of grooming bouts, grooming time |
| ANCOVA | Sex | $F_{1, 13} = 10.05$<br>$p = 0.007$ | $F_{1, 13} = 10.29$<br>$p = 0.007$ |
| | Treatment | $F_{1, 13} = 27.86$<br>$p < 0.001$ | $F_{1, 13} = 27.72$<br>$p < 0.001$ |
| | Sex × treatment | $F_{1, 13} = 4.28$<br>$p = 0.059$ | $F_{1, 13} = 4.19$<br>$p = 0.061$ |

Unlike the original case in Suppl. Tables 3 and 4, nonhomogeneity of regression slopes was not detected. Although analyses with the same multiple sets of covariates except for sex and expansion of two grooming levels were performed, only the results for the optimal set of covariates with respect to BIC are shown for each combination of TD and CC values (in seconds).

**Suppl. Table 9.** The same analyses as for Suppl. Table 7, with different thresholds for detecting non-scratching bouts (TD) and different cutoffs for concatenating separate non-scratching bouts with short intervals (CC) in four 30-minute segments excerpted from the 24-hour behavioral monitoring.

| | | Number of non-scratching bouts | | | | | |
|---|---|---|---|---|---|---|---|
| | | TD-3/CC-6 | TD-1/CC-6 | TD-3/CC-3 | TD-1/CC-3 | TD-3/CC-1 | TD-1/CC-1 |
| ART-ANOVA | Sex | $F_{1, 8} = 21.10$<br>$p = 0.002$ | $F_{1, 8} = 43.91$<br>$p < 0.001$ | $F_{1, 8} = 15.68$<br>$p = 0.004$ | $F_{1, 8} = 21.26$<br>$p = 0.002$ | $F_{1, 8} = 12.39$<br>$p = 0.008$ | $F_{1, 8} = 17.07$<br>$p = 0.003$ |
| | Genotype | $F_{2, 16} = 25.78$<br>$p < 0.001$ | $F_{2, 16} = 32.00$<br>$p < 0.001$ | $F_{2, 16} = 26.12$<br>$p < 0.001$ | $F_{2, 16} = 34.78$<br>$p < 0.001$ | $F_{2, 16} = 27.75$<br>$p < 0.001$ | $F_{2, 16} = 36.25$<br>$p < 0.001$ |
| | Sex × genotype | $F_{2, 16} = 0.86$<br>$p = 0.440$ | $F_{2, 16} = 1.91$<br>$p = 0.180$ | $F_{2, 16} = 1.11$<br>$p = 0.353$ | $F_{2, 16} = 1.98$<br>$p = 0.171$ | $F_{2, 16} = 1.07$<br>$p = 0.365$ | $F_{2, 16} = 2.22$<br>$p = 0.141$ |
| post-hoc tests | *p* -value in pairwise Wilcoxon signed rank test with Bonferroni corrections | KO vs HT 0.006<br>KO vs WT 0.006<br>HT vs WT 0.305 | KO vs HT 0.006<br>KO vs WT 0.006<br>HT vs WT 0.188 | KO vs HT 0.006<br>KO vs WT 0.006<br>HT vs WT 0.598 | KO vs HT 0.006<br>KO vs WT 0.006<br>HT vs WT 0.246 | KO vs HT 0.006<br>KO vs WT 0.006<br>HT vs WT 0.647 | KO vs HT 0.006<br>KO vs WT 0.006<br>HT vs WT 0.246 |

| | | Non-scratching time | |
|---|---|---|---|
| | | TD-3 | TD-1 |
| ART-ANOVA | Sex | $F_{1, 8} = 10.28$<br>$p = 0.013$ | $F_{1, 8} = 10.28$<br>$p = 0.013$ |
| | Genotype | $F_{2, 16} = 14.14$<br>$p < 0.001$ | $F_{2, 16} = 14.14$<br>$p < 0.001$ |
| | Sex × genotype | $F_{2, 16} = 2.00$<br>$p = 0.168$ | $F_{2, 16} = 2.07$<br>$p = 0.159$ |
| post-hoc tests | *p* -value in pairwise Wilcoxon signed rank test with Bonferroni corrections | KO vs HT 0.006<br>KO vs WT 0.082<br>HT vs WT 0.816 | KO vs HT 0.006<br>KO vs WT 0.064<br>HT vs WT 0.697 |

The combination of TD and CC values are shown in seconds.

**Suppl. Table 10.** Two-way ART-ANOVA of number of grooming bouts and grooming time of KO mice in open-field test using sex and treatment as factors.

| Two-way mixed ART-ANOVA | | |
|---|---|---|
| | Grooming time | Number of grooming bouts |
| Sex | $F_{1, 8} = 1.73$<br>$p = 0.225$ | $F_{1, 8} = 1.17$<br>$p = 0.311$ |
| Treatment | $F_{1, 8} = 0.22$<br>$p = 0.649$ | $F_{1, 8} = 0.32$<br>$p = 0.590$ |
| Sex × treatment | $F_{1, 8} = 1.11$<br>$p = 0.324$ | $F_{1, 8} = 0.32$<br>$p = 0.587$ |

**Suppl. Table 11.** Mantel-Haenszel and Woolf tests for independence of grooming levels on treatment groups across sexes.

| Grooming levels | (exact) Mantel-Haenszel test ($p$-value) | Woolf test ($p$-value) |
|---|---|---|
| cluster 1 vs cluster 2 | 1.000 | 0.657 |
| cluster 1-1 vs the others | 1.000 | 0.555 |
| cluster 1-2 vs the others | 0.653 | 0.930 |

The null hypothesis of Mantel-Haenszel test, common odds ratio of grooming levels × treatment groups is one across sexes, was not rejected for all of the cases in which two grooming levels were defined as cluster 1 vs cluster 2, cluster 1-1 vs the others and cluster 1-2 vs the others (see Fig. 4a for definition of clusters). Woolf test did not reject homogeneity of log-odds ratio in all cases. Note that the same results are obtained by permutation of treatment group and sex in these tests.
