# Supplementary material for: Late development of OCD-like phenotypes in Dlgap1 knockout mice
Source: Psychopharmacology (Berl). 2024 Aug 23;242(1):215–31. doi: 10.1007/s00213-024-06668-9 (PMC11742909; doi:10.1007/s00213-024-06668-9)
Supplement: Supplementary file 2 — Supplementary Material 2 [file 213_2024_6668_MOESM2_ESM.pdf]

a **b**

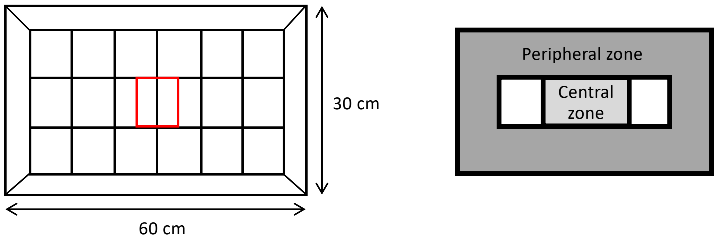

Suppl. Fig. 1. Illustration of open-field arena.
(a) Clear Plexiglas box (60 cm × 30 cm × 20 cm) with 18 (10 cm × 10 cm) squares drawn on its floor. Mice were placed in the center of this arena (the zone indicated by the 10 cm × 10 cm red square).
(b) Central and peripheral zones of the arena are indicated in light gray and dark gray, respectively.

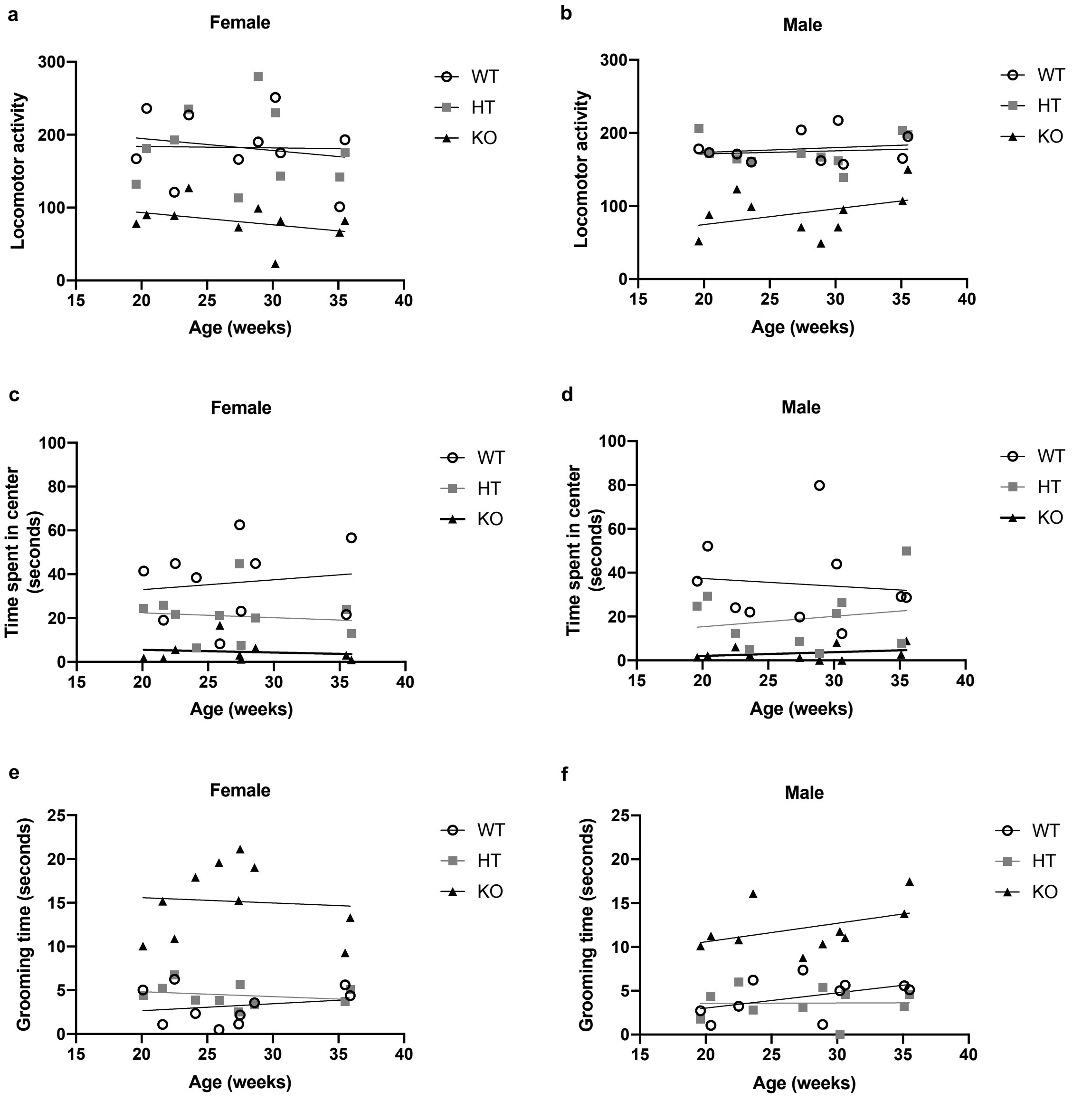

Suppl. Fig. 2 Relationship between age and behavioral parameters measured in open-field test.

The coefficient of age, its standard error and *p*-value in the univariate linear regression of each behavioral parameter in mice of each genotype and sex were as follows: (a) KO, -1.68 ± 1.52, p = 0.30; HT, -0.20 ± 3.28, p = 0.95; WT, -1.65 ± 2.91, p = 0.59, (b) KO, 2.16 ± 1.80, p = 0.26; HT, 0.42 ± 1.32, p = 0.76; WT, 0.66 ± 1.24, p = 0.61, (c) KO, -0.12 ± 0.31, p = 0.70; HT, -0.22 ± 0.71, p = 0.77; WT, 0.45 ± 1.14, p = 0.70, (d) KO, 0.17 ± 0.19, p = 0.21; HT, 0.48 ± 0.88, p = 0.97; WT, -0.35 ± 1.21, p = 0.38, (e) KO, -0.06 ± 0.28, p = 0.83; HT, -0.06 ± 0.08, p = 0.50; WT, 0.08 ± 0.13, p = 0.56, (f) KO, 0.21 ± 0.15, p = 0.21; HT, 0.00 ± 0.11, p = 0.97; WT, 0.12 ± 0.13, p = 0.38. No significant association with age was detected in the above.

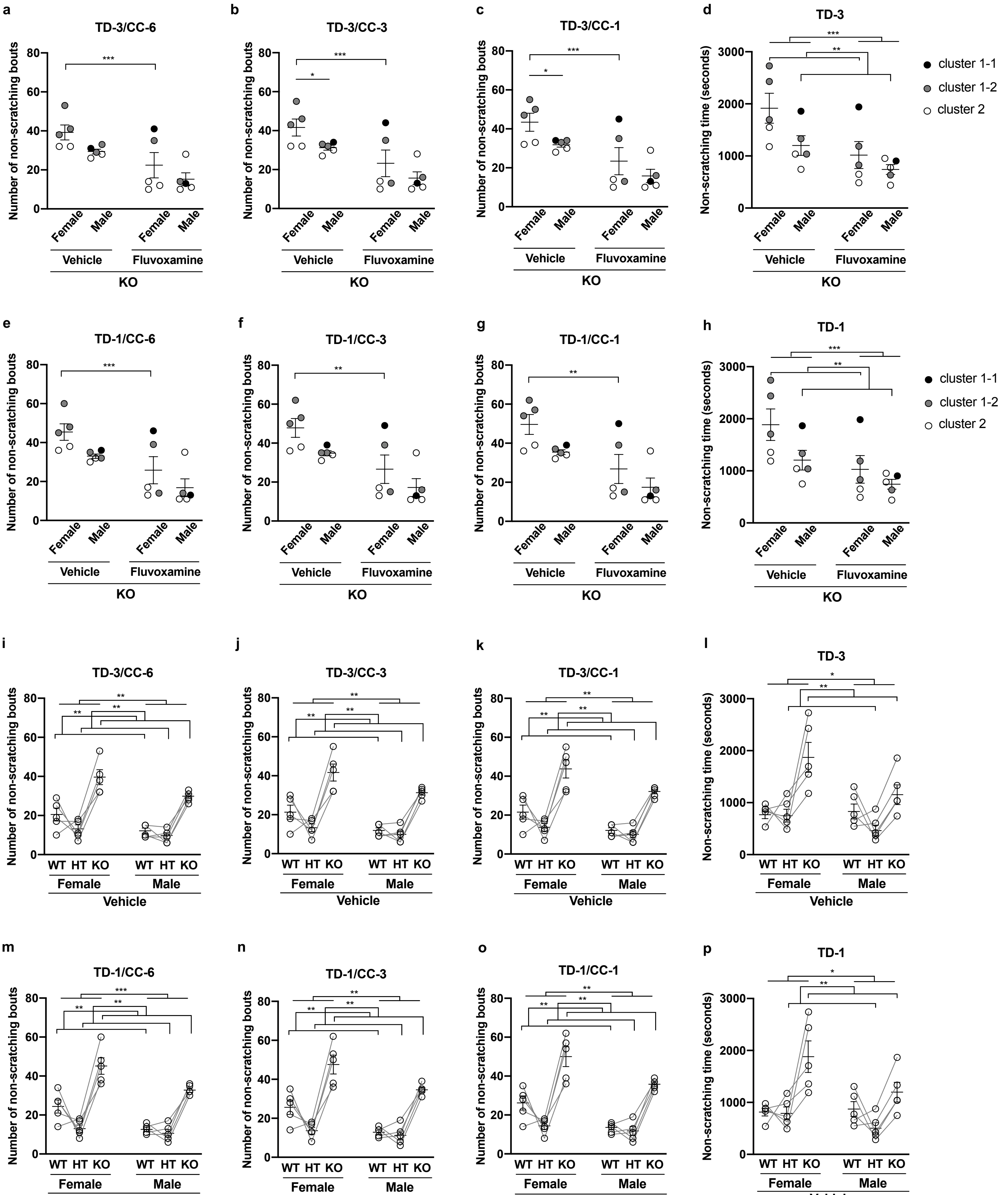

Suppl. Fig. 3. The same analysis as that in Fig. 5 (a)–(f), with different thresholds for detecting non-scratching bouts (TD) and different cutoffs for concatenating separate non-scratching bouts with short intervals (CC) in four 30-minute segments of the 24-hour behavioral monitoring.

For each combination of sex and treatment in KO mice, or for each combination of sex and genotype in vehicle-treated mice, the mean and its standard error of the behavioral parameter indicated in each of panel (a)–(p) are plotted together with data points for individual mice and lines connecting data points for littermates. Values of TD and CC are shown above the graphs (in seconds). Data were analyzed by performing two-way (a)–(h) ANCOVA (using treatment and sex as factors) and (i)–(p) two-way mixed ART-ANOVA (using genotype and sex as factors), whose details are given in Suppl. Tables 8 and 9, respectively. Statistically significant differences confirmed by post-hoc tests are indicated in the panels ($^{***}p < 0.001$; $^{**}p < 0.01$; $^{*}p < 0.05$). Results that did not reach the significance level are not shown in the panel.

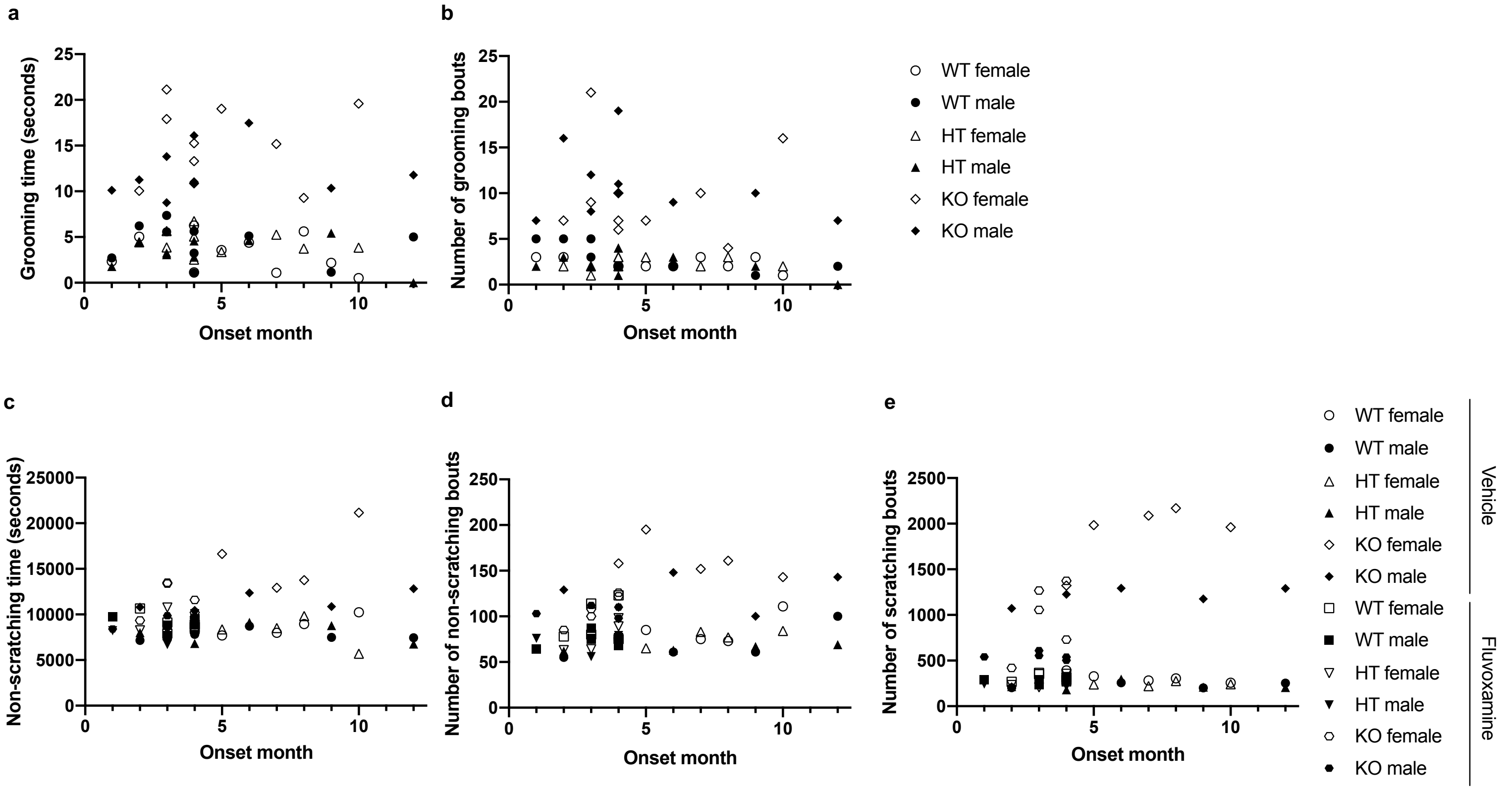

Suppl. Fig. 4. Behavioral parameters vs onset month in open-field test and 24-hour monitoring.

(a)–(e): Grooming time and number of grooming bouts in open-field test, and non-scratching time and numbers of scratching and non-scratching bouts in 24-hour monitoring were plotted by aligning them with the month of onset of skin lesions in the KO littermates. No apparent association between these behavioral parameters and onset month was observed.

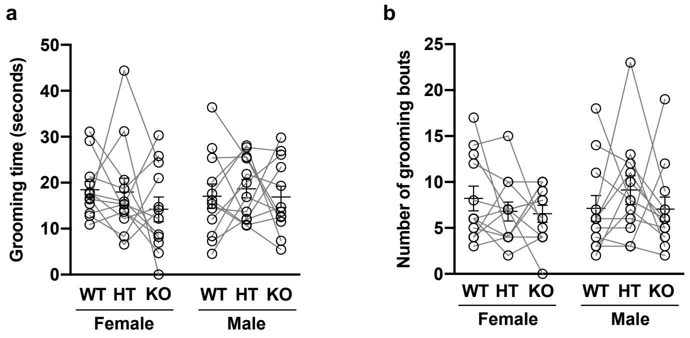

Suppl. Fig. 5. Grooming-related behavioral parameters in SHIRPA primary screen

For each combination of genotype and sex, the mean and its standard error of the behavioral parameter indicated in each of panels, (a) and (b), are plotted together with data points for individual mice and lines connecting data points for littermates. Data were analyzed by performing two-way mixed ART-ANOVA (using genotype and sex as factors). Neither a significant main effect of sex nor genotype, nor their two-way interaction was detected as follows: (grooming time) sex, $F_{1, 22}$ = 0.17, p = 0.686, genotype $F_{2, 44}$ = 0.77, p = 0.471, two-way interaction, $F_{2, 44}$ = 0.64, p = 0.535; (number of grooming bouts) sex, $F_{1, 22}$ = 0.02, p = 0.886, genotype $F_{2, 44}$ = 0.36, p = 0.698, two-way interaction, $F_{2, 44}$ = 0.98, p = 0.383.
